# Toward a Natural Classification of *Botryosphaeriaceae*: A Study of the Type Specimens of *Botryosphaeria sensu lato*

**DOI:** 10.3389/fmicb.2021.737541

**Published:** 2021-11-03

**Authors:** Ying Zhang, Yupei Zhou, Wei Sun, Lili Zhao, D. Pavlic-Zupanc, Pedro W. Crous, Bernard Slippers, Yucheng Dai

**Affiliations:** ^1^School of Ecology and Nature Conservation, Beijing Forestry University, Beijing, China; ^2^Department of Microbiology, Faculty of Natural and Agricultural Sciences, DST-NRF Centre of Excellence in Tree Health Biotechnology, Forestry and Agricultural Biotechnology Institute, University of Pretoria, Pretoria, South Africa; ^3^Westerdijk Fungal Biodiversity Institute, Utrecht, Netherlands; ^4^Department of Genetics, Faculty of Natural and Agricultural Sciences, DST-NRF Centre of Excellence in Tree Health Biotechnology, Forestry and Agricultural Biotechnology Institute, University of Pretoria, Pretoria, South Africa

**Keywords:** *Botryosphaeriales*, phylogeny, sexual stage, taxonomy, type specimens

## Abstract

The genus *Botryosphaeria* includes more than 200 epithets, but only the type species, *Botryosphaeria dothidea* and a dozen or more other species have been identified based on DNA sequence data. The taxonomic status of the other species remains unconfirmed because they lack either morphological information or DNA sequence data. In this study, types or authentic specimens of 16 “*Botryosphaeria*” species are reassessed to clarify their identity and phylogenetic position. *nu*DNA sequences of four regions, ITS, LSU, *tef1-α* and *tub2*, are analyzed and considered in combination with morphological characteristics. Based on the multigene phylogeny and morphological characters, *Botryosphaeria cruenta* and *Botryosphaeria hamamelidis* are transferred to *Neofusicoccum*. The generic status of *Botryosphaeria aterrima* and *Botryosphaeria mirabile* is confirmed in *Botryosphaeria*. *Botryosphaeria berengeriana* var. *weigeliae* and *B*. *berengeriana* var. *acerina* are treated synonyms of *B. dothidea*. *Botryosphaeria mucosa* is transferred to *Neodeightonia* as *Neodeightonia mucosa*, and *Botryosphaeria ferruginea* to *Nothophoma* as *Nothophoma ferruginea*. *Botryosphaeria foliicola* is reduced to synonymy with *Phyllachorella micheliae*. *Botryosphaeria abuensis*, *Botryosphaeria aesculi*, *Botryosphaeria dasylirii*, and *Botryosphaeria wisteriae* are tentatively kept in *Botryosphaeria sensu stricto* until further phylogenetic analysis is carried out on verified specimens. The ordinal status of *Botryosphaeria apocyni*, *Botryosphaeria gaubae*, and *Botryosphaeria smilacinina* cannot be determined, and tentatively accommodate these species in *Dothideomycetes incertae sedis*. The study demonstrates the significance of a polyphasic approach in characterizing type specimens, including the importance of using of DNA sequence data.

## Introduction

*Botryosphaeria* Ces. and De Not. was formally established by Cesati and De Notaris (1863) based on 12 species, but the generic type was not designated, neither were detailed descriptions provided for these species. Furthermore, *Botryosphaeria* was discussed as being heterogeneous, and considered to probably represent three genera, i.e., *Botryosphaeria*, *Gibberella* Sacc. and *Lisea* Sacc. (Cesati and De Notaris, 1863). Subsequently, De Notaris (1863) described four additional species in *Botryosphaeria*, viz., *B. berengeriana* De Not., *Botryosphaeria dispersa* De Not., *Botryosphaeria juglandina* De Not., *Botryosphaeria moricola* Ces. and De Not. Saccardo (1877) emended the generic description of Cesati and De Notaris (1863) to exclude hypocreaceous species, and formally introduced *Gibberella* and *Lisea* to accommodate them. von Höhnel (1909) designated *B. berengeriana* as the generic type. Based on Saccardo’s (1877) amendment, Theissen and Sydow (1915) suggested *Botryosphaeria quercuum* (Schwein.) Sacc. as the generic type, which was supported by von Arx and Müller (1954). However, neither *B*. *berengeriana* nor *B*. *quercuum* had been included in the original description of *Botryosphaeria*. By 1954, more than 100 species had been described in *Botryosphaeria*. von Arx and Müller (1954) reduced 108 taxa to synonymy with *B*. *quercuum*, and 24 taxa with *Botryosphaeria dothidea* (Moug.) Ces. and De Not. Only 11 species remained accepted in *Botryosphaeria*. However, the sexual morph on which these synonymies were known to be morphologically conserved, and the treatment was therefore not widely accepted (Shoemaker, 1964; Sivanesan, 1984; Slippers et al., 2004). Barr (1972) proposed *B*. *dothidea* as the lectotype of *Botryosphaeria*, since it conformed to Saccardo’s (1877) amendment of the genus, and was one of the original species described in *Botryosphaeria* by Cesati and De Notaris (1863). This typification has subsequently been widely accepted (Smith et al., 2001; Slippers et al., 2004; Phillips et al., 2013).

Early researchers described *Botryosphaeria* species mostly based on their sexual morphs and host associations, which led to the addition of numerous species (Cesati and De Notaris, 1863; De Notaris, 1863; Saccardo, 1877, 1882; Grossenbacher and Duggar, 1911; Putterill, 1919; Trotter, 1928). Currently, more than 200 epithets are included in *Botryosphaeria* (November 2020)^1^, and the genus is considered as being heterogeneous (Slippers et al., 2004; Crous et al., 2006; Phillips et al., 2008, 2013).

Slippers et al. (2004) designated an epitype for *B*. *dothidea*, with a modified description, ex-type culture and DNA sequence data, which shed light on the circumscription of *Botryosphaeria sensu stricto*. Based on a LSU phylogeny, Crous et al. (2006) identified 10 phylogenetic lineages in *Botryosphaeria sensu lato*, which corresponded to different asexual genera. Using a phylogeny based on the analyses of sequence data for five loci (SSU, LSU, ITS, *tub2*, and *tef1-α*), Phillips et al. (2008) clarified the morphology of several genera in the *Botryosphaeriaceae*, and introduced two new genera, i.e., *Barriopsis* A.J.L. Phillips, A. Alves and Crous and *Spencermartinsia* A.J.L. Phillips, A. Alves and Crous. Furthermore, Phillips et al. (2013) recognized seven species in *Botryosphaeria sensu stricto*, i.e., *Botryosphaeria agaves* (Henn.) E.J. Butler, *Botryosphaeria corticis* (Demaree and Wilcox) Arx and E. Müll., *B*. *dothidea*, *Botryosphaeria fabicerciana* (S.F. Chen, Pavlic, M.J. Wingf. and X.D. Zhou) A.J.L. Phillips and A. Alves, *Botryosphaeria fusispora* Boonmee, Jian K. Liu and K.D. Hyde, *Botryosphaeria ramosa* (Pavlic, T.I. Burgess and M.J. Wingf.) A.J.L. Phillips and A. Alves and *Botryosphaeria scharifii* Abdollahz., Zare and A.J.L. Phillips. Subsequently, a few more species of *Botryosphaeria*, e.g., *Botryosphaeria auasmontanum* F.J.J. Van der Walt Slippers and G.J. Marais, *Botryosphaeria minutispermatia* Ariyawansa, K.D. Hyde and Z.Y. Liu, *Botryosphaeria qingyuanensis* G.Q. Li and S.F. Chen, *Botryosphaeria sinensia* Y.P. Zhou and Y. Zhang ter. and *Botryosphaeria rosaceae* Y.P. Zhou and Y. Zhang ter. were described (Slippers et al., 2014; Ariyawansa et al., 2016; Dissanayake et al., 2016; Zhou et al., 2016, 2017; Li et al., 2017). Zhang et al. (2021) reviewed the species within *Botryosphaeriales*, and accepted eight species within *Botryosphaeria sensu stricto*, *viz*., *B. agaves, B. corticis, B. dothidea, B. fabicerciana, Botryosphaeria kuwatsukai, B. qingyuanensis, B. ramosa*, and *B. scharifii*. To date, however, the taxonomic status of most taxa accommodated in *Botryosphaeria sensu lato* remains uncertain.

As a fundamental element in the current Code of Nomenclature for algae, fungi, and plants, type studies play a critical role in epitypification, as well as in defining species or genera of *Ascomycetes* (Zhang et al., 2009). Specifically for taxa in the *Botryosphaeriales*, there are few studies based on DNA sequence data. Almost all the older names linked to *Botryosphaeria* lack cultures or DNA sequence data, and they can consequently not be classified to genus or even family rank with confidence. Thus, these names are unusable unless they are either epitypified or supplemented with DNA sequence data (Slippers et al., 2014). The aims of the present study were thus to verify the identity of 17 selected type or authentic specimens (representing 16 species) currently placed in *Botryosphaeria*, using morphological characteristics and *nu*DNA sequence data.

## Materials and Methods

### Type Study

Type specimens of 16 putative *Botryosphaeria* species were obtained on loan from the Conservatoire et Jardin botaniques de la Ville de Genève (G), Naturhistorisches Museum Wien (W), Field Museum of Natural History (F), Royal Botanic Gardens (K), University of Michigan (MICH) and New York State Museum (NYS) (Table 1). The type specimens were described and illustrated following the protocol by Zhang et al. (2012). Sections made from specimens were studied at × 1,000 magnification using a Nikon E600 compound microscope. Ascomata were examined under a Leica M125 dissecting microscope. Sections of ascomata, hamathecia, asci, and ascospores were mounted in water or 10–100% lactic acid. Micrographs were made from tissues mounted in water with 10–100% lactic acid or cotton blue. Question marks (?) indicate possible type specimens.

**TABLE 1:** List of the herbarium specimens characterized in this study.

Current name	Basionym	Herbarium number	DNA fragments obtained
***Species included in Botryosphaeriaceae***			
*Botryosphaeria aterrima*	*Melanops aterrima*	G 00266252	ITS/LSU/*tef1-α*
*B. dothidea*	*B. berengeriana var*. *acerina*	F C0003484F	—
*B*. *dothidea*	*B. berengeriana var. weigeliae*	MICH 13862	ITS/*tub2*
*B*. *mirabile*	*M. mirabilis*	G 00266251	ITS/LSU
*Neodeightonia mucosa*	*B. mucosa*	IMI 204341	—
*Neofusicoccum cruenta*	*M. cruenta*	W 1978-0010992/24018	LSU
*N. hamamelidis*	*B. hamamelidis*	W 07238/29850	ITS/*tef1-α*/*tub2*
***Species tentatively accommodate in Botryosphaeria***			
? *B*. *abuensis*	*B. abuensis*	IMI 192142	—
? *B*. *aesculi*	*Laestadia aesculi*	NYS f93	—
? *B*. *dasylirii*	*Dothidea dasylirii*	NYS f950	—
? *B*. *wisteriae*	*Thuemenia wisteriae*	MICH 15081	—
**Taxa excluded from *Botryosphaeriales***			
*Nothophoma ferruginea*	*M. ferruginea*	G 00127285	ITS/LSU
*Phyllachorella micheliae*	*B. foliicola*	IMI 316002	—
***Dothideomycetes incertae sedis***			
*B. gaubae*	*B. gaubae*	W 1992-05937	—
*Laestadia apocyni*	*Laestadia apocyni*	MICH 14281	—
*Sphaeria smilacinina*	*Sphaeria smilacinina*	NYS f2818	—

### *nu*DNA Extraction, PCR Amplification and Cloning

After getting the DNA extraction permission, *nu*DNA was extracted from ascomata or conidiomata using a Forensic DNA kit (OMEGA Bio-tek). The internal transcribed spacer of regions (1 and 2) of the *nu*DNA (ITS) was amplified and sequenced with primers ITS-4 and ITS-5 (White et al., 1990). The 28S large subunit *nu*DNA (LSU) was amplified and sequenced with primers LROR and LR5 (Vilgalys and Hester, 1990). Sections of the translation elongation factor-1α (*tef1-α*) with primers EF1-688F and EF1-1251R (Alves et al., 2008) and the β-tubulin gene (*tub2*) with primers Bt2a and Bt2b (Glass and Donaldson, 1995). PCR amplification and sequencing was conducted following the protocol by Zhang et al. (2009). Some of the resulting sequences had ambiguous base calls, possibly due to the contamination of the other fungi occurring on the specimens. All PCR products exhibiting this phenomenon were cloned using the pGEM-T Vector System I cloning kit (Promega).

### Sequence Alignment and Phylogenetic Analysis

For the sequences obtained, a search was conducted using BLAST (Basic Local Alignment Search Tool) in GenBank sequences^2^ to confirm the generic status of the related specimens. Sequence data for each individual gene region, ITS, LSU, *tef1-α* and *tub2*, as well as the combined datasets were used to infer the phylogenetic relationships among all confirmed *Botryosphaeria*, *Neofusicoccum*, *Nothophoma* species for which sequence data were available from GenBank (see text footnote 2), together with the sequences generated in this study. Alignments were made in MEGA v. 6 (Tamura et al., 2013) and phylogenetic analyses performed in PAUP v. 4.0b10 (Swofford, 2002) and MrBayes v. 3.1.2 (Ronquist and Huelsenbeck, 2003). Prior to phylogenetic analyses, ambiguous sequences at the start and the end of sequences were deleted and gaps manually adjusted to optimize the alignments. Maximum Parsimony (MP) was used to conduct heuristic searches as implemented in PAUP with the default options method (Zhang et al., 2008). Analyses were made under different parameters of maximum parsimony criteria as outlined in Zhang et al. (2008). Clade stability was assessed in a bootstrap analysis with 1,000 replicates, random sequence additions with MaxTrees set to 1,000 and other default parameters as implemented in PAUP. Maximum likelihood (ML) was also conducted using heuristic searches with the default options method as implemented in PAUP. For the ML analysis, best-fit model of nucleotide evolution was selected by hierarchical likelihood ratio test (hLRT) in MrModeltest 2.3. A bootstrap analysis with 1,000 replicates was used to test the statistical support of the branches. For the MrBayes analyses, the best-fit model of nucleotide evolution was selected by Akaike information criterion (AIC; Posada and Buckley, 2004) in MrModeltest v. 2.3. The metropolis-coupled Markov Chain Monte Carlo (MCMCMC) approach was used to calculate posterior probabilities (Huelsenbeck and Ronquist, 2005). Trees were viewed in TREEVIEW. Phylograms obtained based on combined loci or for a single locus were all deposited in TreeBASE. The nucleotide sequences reported in this study were deposited in GenBank (Supplementary Table 1).

## Results

### Molecular Phylogenetic Analysis

Based on the results of BLAST in GenBank, *Botryosphaeria aterrima* (Fuckel) Sacc., *B*. *berengeriana* var. *weigelae* Rehm and *Botryosphaeria mirabile* (Fuckel) Cooke belong to *Botryosphaeria*, *B. cruenta* and *B*. *hamamelidis* to *Neofusicoccum*, *Botryosphaeria ferruginea* to *Nothophoma* Qian Chen and L. Cai. The phylogenetic analysis of the *Botryosphaeria* dataset included 14 ingroup taxa and two outgroup taxa (Supplementary Figure 4). The combined ITS, LSU, *tef1-a*, and *tub2* matrix contained 2,268 characters, of which 1,940 were constant and 39 were variable and parsimony-uninformative. Maximum parsimony analysis of the remaining 289 parsimony-informative characters resulted in 2,275 equally most parsimonious trees (Supplementary Figure 4). The phylogenetic tree resulting from the Bayesian analysis using the general time reversible model of *nu*DNA evolution (Rodríguez et al., 1990), including estimation of non-variable sites and assuming a discrete gamma distribution with six rate categories (GTR+Γ+G), had a topology identical to the MP tree presented. In both analyses (MP and Bayesian), the clade of *Botryosphaeria* had a high bootstrap support (100% for MP) and high posterior probabilities (1.00 for MrBayes). The phylogenetic status of three species *B*. *agaves* (Henn.) E.J. Butler, *B*. *ramosa* and *B*. *scharifii* was resolved in a well-supported clade with *B*. *agaves* basal to all other species of *Botryosphaeria.* The phylogenetic relationships among *B*. *aterrima*, *B*. *auasmontanum*, *B*. *berengeriana* var. *weigeliae* Rehm, *B*. *corticis*, *B*. *dothidea*, *B*. *fabicerciana*, *B*. *fusispora*, *B*. *minutispermatia*, *B*. *mirabile*, *B*. *rosaceae*, and *B*. *sinensia* could not be resolved (Supplementary Figure 4, TreeBASE number S21054).

The analysis for *Neofusicoccum* involved 38 taxa including two outgroup species, i.e., *B. corticis* and *B*. *dothidea*. The combined ITS, *tef1-α* and *tub2 nu*DNA sequence matrix included 920 characters, 136 were constant and 39 were variable and parsimony-uninformative. Maximum parsimony analysis for the remaining 181 parsimony-informative characters resulted in 5,000 equally most parsimonious trees (Supplementary Figure 5, TreeBASE number S21059). The phylogenetic tree resulting from the Bayesian analysis using the general time reversible model of *nu*DNA evolution (Rodríguez et al., 1990), including estimation of invariable sites and assuming a discrete gamma distribution with six rate categories (GTR+Γ+G), had a topology identical to the MP tree presented. In both analyses (MP and Bayesian) the clade accommodating *Neofusicoccum* had a high level of support (100% for MP and 1.00 PP for MrBayes). Isolates of *N*. *hamamelidis* formed sub-clade representing an individual species of *Neofusicoccum* (Supplementary Figure 5).

The analysis for *Neofusicoccum* LSU sequences included 18 taxa with two outgroup species, *B. corticis* and *B*. *dothidea*. The LSU *nu*DNA sequence dataset contained 847 characters, of which 779 were constant and 42 were variable and parsimony-uninformative. Maximum parsimony analysis of the remaining 26 parsimony-informative characters resulted in 22 equally most parsimonious trees (Supplementary Figure 6, TreeBASE number S21050). *Neofusicoccum cruenta* and *N. hamamelidis* formed a sub-clade representing an individual species, respectively, while lacked of support (Supplementary Figure 6).

The analysis for *Nothophoma* spp. involved 22 taxa including one outgroup species, i.e., *Didymella calidophila*. The combined ITS and LSU sequence matrix included 1,821 characters, of which 1,751 were constant and 38 were variable and parsimony-uninformative. Maximum parsimony analysis for the remaining 32 parsimony-informative characters resulted in 1,000 equally most parsimonious trees. For the Bayesian analysis, TNe+I was selected as the best-fit model for the ITS and LSU dataset, had a topology identical to the MP tree and ML tree presented. Phylogenetically, species of *Nothophoma* formed a robust clade. Isolates of *No*. *ferruginea* formed sub-clade representing an individual species of *Nothophoma* spp. Only the Bayesian tree is presented herein with MP, PP, and ML values plotted on the branches (Supplementary Figure 7).

### Taxonomy

#### Species Included in *Botryosphaeriaceae*

**FIGURE 1:**
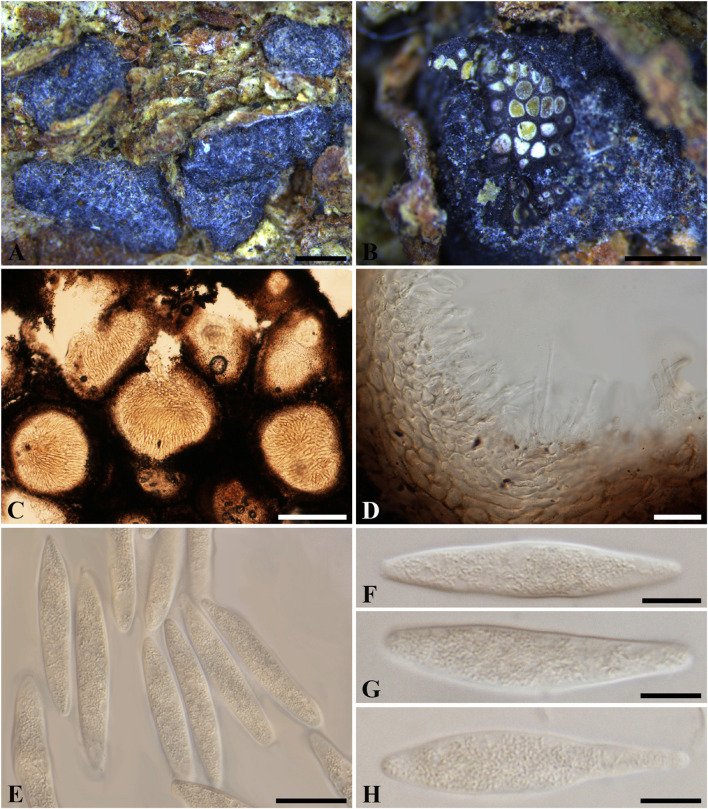
***Botryosphaeria aterrima*** (G 00266252, **holotype**). **(A)** Dense botryose cluster of conidiomata erumpent through the bark. **(B)** Transverse section of the conidiomata. **(C)** Longitudinal section of conidiomata. **(D)** Conidiogenous cells and paraphyses. **(E–H)** Conidia. Scale bars: **(A)** = 5 mm, **(B)** = 1 mm, **(C)** = 200 μm, **(D,E)** = 20 μm, **(F–H)** = 10 μm.

***Botryosphaeria aterrima*** (Fuckel) Sacc. Syll. fung. (Abellini) 1: 458 (1882). Figure 1

≡ *Melanops aterrima* Fuckel, Jb. nassau. Ver. Naturk. 23–24: 225 (1870) [1869–1870]

*Ascostromata* not observed. *Conidiostromata* forming dense botryose aggregate, 2–7 mm diam., pseudothecial, aggregated into botryose clusters, 220–420 μm diam., spherical to globose with a central ostiole, ½ to ¾ emergent, rarely embedded, black. *Peridium* comprising 7–15 layers of *textura angularis*, outer region of dark brown cells, inner region of 3–7 layers of pale brown cells lining the locule. *Paraphyses* when present hyaline, septate, up to 70 μm long, 2–4 μm broad at the base, tapering to acutely rounded apices, 1.5–2 μm broad at the tip. *Conidiogenous cells* holoblastic, hyaline, sub-cylindrical, 8–20 × 3–5 μm. *Conidia* hyaline, narrowly fusiform, or irregularly fusiform, base subtruncate to bluntly rounded, (40–)42–60(–62) × (7–)9–11 μm (−x = 51.3 × 9.9 μm, *n* = 20), L/W = 5.2.

Specimen examined – GERMANY, Hessen, *Ulmus* sp. (*Ulmaceae*), Fuckel, K.W.G. (G 00266252, **holotype**).

Notes – Although phylogenetic analysis based on combined loci of ITS, *tef1-a* and LSU confirmed *Botryosphaeria aterrima* within *Botryosphaeria*, it cannot be distinguished from *B*. *auasmontanum*, *B*. *berengeriana* var. *weigeliae*, *B*. *dothidea*, *B*. *minutispermatia*, and *B*. *mirabile* (Supplementary Figure 4). The two isolates of *B*. *dothidea* used in this study are ex-epitype (CBS 115476) and a verified isolate (CBS 110302), respectively (Slippers et al., 2004; Phillips et al., 2013). *Botryosphaeria dothidea* is known as a cosmopolitan species associated with woody plants in numerous families (Slippers et al., 2004; Marsberg et al., 2017). The type of *B*. *aterrima* reassessed here was collected from *Ulmus* sp. in Germany, and only the asexual morph was observed. Conidia of *B*. *aterrima* examined in this study were much larger [(40–)42–60(–62) × (7–)9–11 μm] than those of *B*. *dothidea* [(17–)18–20(–22) × 4–5 μm, as reported by Slippers et al. (2004), Table 2]. A remarkable feature of *B. aterrima* was its multiloculate conidiomata, which was comparable with members of *Aplosporellaceae* and *Melanopsaceae*. The hyaline, fusiform conidia lacking mucous sheath, however, differed from these two families. Thus, we treat *B*. *aterrima* as a separate species within *Botryosphaeria sensu stricto*.

**TABLE 2:** Morphological characteristics of *Botryosphaeria* spp. surely assigned within *Botryosphaeria* so far.

Items	*B. agaves*	*B. aterrima*	*B. corticis*	*B. dothidea*	*B. fabicerciana*	*B. kuwatsukai*	*B. mirabile*	*B. qingyuanensis*	*B. ramosa*	*B. scharifii*
**Ascomata**										
Size (μm)	600–800	UN	up to 250	200-500	UN	UN	UN	UN	UN	UN
**Pseudoparaphyses within ascomata**										
Width	3–5	UN	UN	2–4	UN	UN	UN	UN	UN	UN
Septum	AS	UN	S	S	UN	UN	UN	UN	UN	UN
**Asci**										
Size (μm)	91–122 × 27–38	UN	145–165 × 25–28	63-125 × 16–20	UN	UN	UN	UN	UN	UN
**Ascospores**										
Size (μm)	21–43 × 8–12	UN	24–34.5 × 9.5–13.5	17–32 × 6–10	UN	UN	10 × 5	UN	UN	UN
L/W	UN	UN	2.5	2.9	UN	UN	UN	UN	UN	UN
Sheath	WMS	UN	UN	UN	UN	UN	UN	UN	UN	UN
**Conidiomata**										
Size (μm)	UN	220–420	up to 450	200-500	245–525	UN	UN	up to 317 × 229	up to 510	up to 760
Papilla	UN	up to 70	UN	up to 110	UN	UN	UN	UN	UN	UN
**Paraphyses within conidiomata**										
ATB	UN	2–4	UN	2.5–6	UN	UN	UN	UN	UN	UN
ATT	UN	1.5–2	UN	2–2.5	UN	UN	UN	UN	UN	UN
**Conidiophores**										
Size (μm)	UN	UN	7.5–14 × 3.5–4.5	23–35 × 4–5	UN	UN	UN	UN	UN	7.5–33.5 × 2–4.5
**Conidiogenous cells**										
Size (μm)	UN	8–20 × 3–5	12.5–17.5 × 2.5–4.5	6–20 × 2–5	6.5–16 × 2–4.5	7–18 × 2–4	UN	(7–)7.5–12(–14.5) × (2–)2.5–3.5	6–11 × 2–3.5	7–15 × 1.5–3.5
Septum	UN	AS	AS	RS	RS	UN	UN	UN	AS	AS
**Conidia**										
Size (μm)	UN	40–62 × 7–11	20.5–34.5 × 5–7.5	17–34 4–7.5	16.5–26 × 4.5–7.5	(18.5–)20–24.5(-26) × 5–7(-8)	UN	(15–)19.5–24.5 (–28.5) × (5–)6–6.5(–7.5)	11–16 × 4.7–7	11.5–19 × 4–6.5
L/W	UN	5.2	4.5	4.9	3.8	3.6	UN	3.5	2.3	2.7
Pigment	UN	H	H	HS	H	H	UN	H	H	H
Sheath	UN	UN	WMS	UN	UN	UN	UN	UN	UN	UN
**Spermatogenous cells**										
Size (μm)	UN	UN	14.5–20.5 × 1.5–2.3	7–10 × 2–3	UN	3–10 × 1–2	UN	UN	UN	UN
**Spermatia**										
Shape	UN	UN	R	A to R	UN	A to R	globose	UN	UN	UN
Size (μm)	UN	UN	4–6 × 1.5–2	3-6 × 1.5-2	UN	3–8 × 1–2	3-4?	UN	UN	UN
**Hosts**	*Agaves* sp.	*Ulmus* sp.	*Vaccinium* spp.	WIF	*Eucalyptus* sp.	*Malus* sp. *Pyrus* sp.	*Quercus* sp.	*Eucalyptus* sp.	*Eucalyptus camaldulensis*	*Mangifera indica*
**Reference**	Liu et al., 2012	this study	Phillips et al., 2013	Slippers et al., 2004	Chen et al., 2011	Xu et al., 2015	Fuckel, 1870, this study	Li et al., 2017	Phillips et al., 2013	Abdollahzadeh et al., 2013

*AS, aseptate; S, septate; RS, rarely septate and becoming 1–2 septa before germination or after being discharged from the pycnidium or with age; UN, unknown; L, Length; W, Width; H, Hyaline; HS, Hyaline, sometimes dark-walled with age; WMS, with mucous sheath; R, rod-shaped; A, allantoid; ATB, Width of paraphyses at the base; ATT, width of paraphyses at the tip.*

**FIGURE 2:**
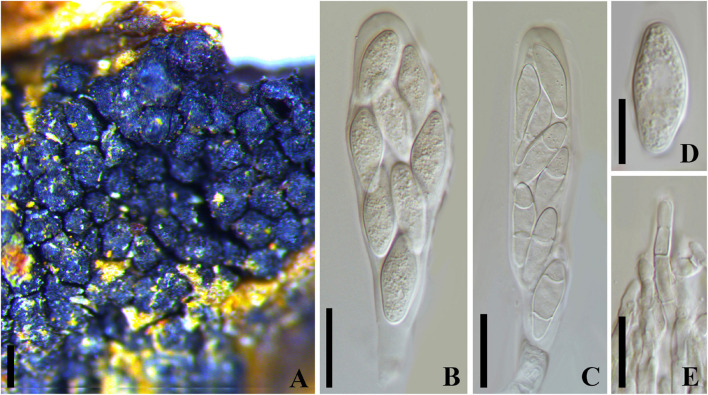
***Botryosphaeria dothidea*** (MICH 13862, **isotype** of *B*. *berengeriana* var. *weigeliae*). **(A)** Botryose clusters of ascomata erumpent through twig epidermis. **(B,C)** Asci with hyaline aseptate or septate ascospores. **(D)** Hyaline ascospore. **(E)** Hyaline, septate, and cellulous pseudoparaphyses. Scale bars: **(A)** = 200 μm, **(B,C)** = 20 μm, **(D,E)** = 10 μm.

***Botryosphaeria dothidea*** (Moug. : Fr.) Ces. and De Not., Comment. Soc. Crittog. Ital. 1:212. 1863. Figure 2

= *Sphaeria dothidea* Moug.: Fr. in Fries, Syst. Mycol. 2:423. 1823
= *Botryosphaeria berengeriana* De Not., Sfer. Ital. 82.1863[1864]
= *Botryosphaeria berengeriana* var. *acerina* Rehm, Annls mycol. 7(6): 533 (1909)
= *Botryosphaeria berengeriana* var. *weigeliae* Rehm, Annls mycol. 12(2): 168 (1914)

*Ascostroma* erumpent to nearly superficial, 1.5-4.5 mm diam. *Ascomata* 110-240 μm diam., pseudothecial, forming botryose clusters of up to 50 locules, globose with a central ostiole, papillate or not, black. *Peridium* comprising 6–15 layers of *textura angularis*, outer region of dark brown cells, inner region of 2–3 layers of hyaline cells lining the locule (Slippers et al., 2004). *Pseudoparaphyses* filiform, cellular, septate, 3–4 μm wide. *Asci* 8-spored, bitunicate, cylindric-clavate to clavate with a short pedicel, 72-130 × 17–27 μm, forming among pseudoparaphyses. *Ascospores* hyaline, broadly fusoid to ellipsoidal, smooth or with granular contents, sometimes become 1–2 septa with age, biseriate in the ascus, (18–)20–26(–28) × 7–10 μm (−x = 23.3 × 8.8 μm, *n* = 20), L/W = 2.6. *Spermatia* not observed.

Specimen examined – RUSSIA, Batum (i), Caucasus, on cortex *Weigela* sp. (*Caprifoliaceae*), Newodowski (MICH 13862, **isotype** of *B. berengeriana* var. *weigeliae*). United States, Washington, on bark of *Acer macrophyllum* (*Aceraceae*), June 1906, S. A. Harper (F C0003484F, **holotype** of *Botryosphaeria berengeriana var. acerina*).

Notes – The phylogeny based on ITS and *tub2 nu*DNA sequence analysis indicated that *Botryosphaeria berengeriana* var. *weigeliae* and *B*. *dothidea* cluster together with ITS [only one base-pair differences (of 202 base-pairs) and *tub2* totally identical (of 344 base-pairs), Supplementary Figure 4]. All the morphological characteristics of *B*. *berengeriana* var. *weigeliae* agree with *B*. *dothidea* but the 1–2-septate mature ascospores (Slippers et al., 2004; Figure 2), which is insufficient to separate a species. Thus, we treated *B*. *berengeriana* var. *weigeliae* a synonym of *B. dothidea*. In addition, *B*. *berengeriana* var. *acerina* was reduced to synonymy with *B*. *dothidea* because of their morphological similarities.

**FIGURE 3:**
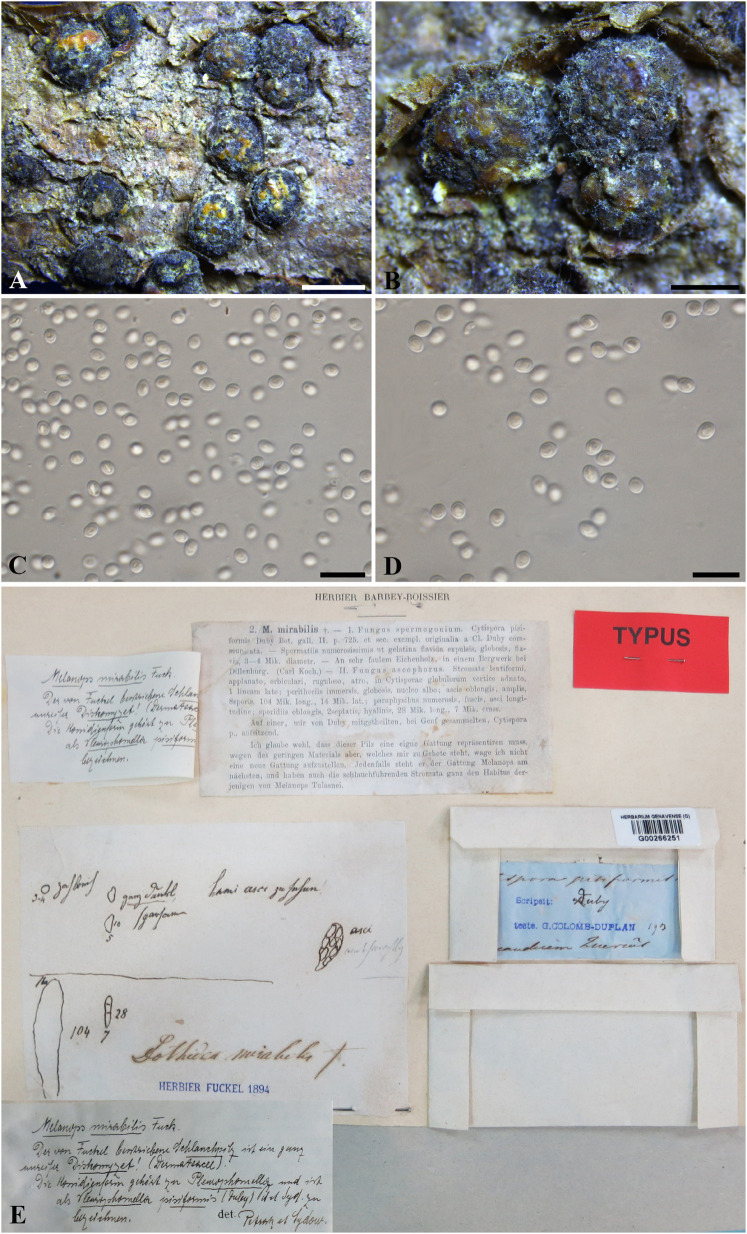
***Botryosphaeria mirabile*** (G 00266251, **holotype**). **(A,B)** Fruiting bodies erumpent through the epidermis of twigs. **(C,D)** Spermatia produced in the fruiting bodies. **(E)** Herbarium label. Scale bars: **(A)** = 2 mm, **(B)** = 1 mm, **(C,D)** = 10 μm.

***Botryosphaeria mirabile*** (Fuckel) Cooke, Grevillea 13 (no. 68): 108 (1885). Figure 3

≡ *Melanops mirabilis* Fuckel, Jahrb. Nassauischen Vereins Naturk. 23–24: 225 (1870)

*Ascostromata* erumpent, clustered, black, with yellow globules on the top, 1–2 mm diam., globose, gregarious. *Asci* 8-spored, bitunicate, broadly clavate. *Pseudoparaphyses* not observed. *Ascospores* fusiform, irregularly biseriate to triseriate in asci, up to 10 × 5 μm. *Spermatia* numerous, ellipsoid to subglobose, hyaline, 3–4 μm (data of sexual stage are obtained from the label of the type specimen).

Specimen examined – SWITZERLAND, Genève, on trunk of *Quercus* sp. (*Fagaceae*), J.E. Duby (G 00266251, **holotype**).

Notes – *Melanops mirabilis* was introduced by Fuckel (1870), and was assigned to *Botryosphaeria* as *B*. *mirabilis* by Cooke (1885). Some large spermatogonia (up to 5 mm diam.) were found on the type specimen examined in this study, which contained numerous subglobose, pale brown spermatial-like cells (*ca*. 3–5 μm diam.) (Figure 3), from which the *nu*DNA was extracted in this study. Some immersed ascomata were also sectioned, but they were in ordinately old to enable a morphological study. Both spermatia and the sexual morph were considered in the original description (Fuckel, 1870). The sexual morph was described as having immersed pseudothecia, numerous, dark brown pseudoparaphyses, narrowly clavate asci, and oblong, hyaline, 2-septate ascospores which disagree with the concept of *Botryosphaeria*. Two distinct sexual morphs were illustrated on the envelope of the type specimen (Figure 3). Besides the one mentioned above, the other was illustrated as having broadly clavate asci, hyaline, aseptate ascospores (10 × 5 μm), which fits the concept of *Botryosphaeria* (Figure 3). Thus, the second illustrated sexual morph corresponds with *B*. *mirabile* (Figure 3). *nu*DNA sequence comparisons based on ITS and LSU indicated that *Melanops mirabilis* resides in *Botryosphaeria* (Supplementary Figure 4), although it cannot be distinguishable from *B*. *aterrima*, *B*. *auasmontanum*, *B*. *berengeriana* var. *weigelae*, *B*. *dothidea*, and *B*. *minutispermatia.* In addition, *B*. *mirabile* differs from *B*. *dothidea* by its *nu*DNA loci, e.g., 6 bp differences in ITS (1%) and 5 bp in LSU (1.2%). Based on its subglobose pale brown spermatia, small-sized ascospores as well as its unique *nu*DNA loci, we retain *B*. *mirabile* as a separate species in *Botryosphaeria sensu stricto*.

***Neodeightonia mucosa*** (S.J. Kaur) Y. Zhang ter and Y.P. Zhou, comb. nov.

**FIGURE 4:**
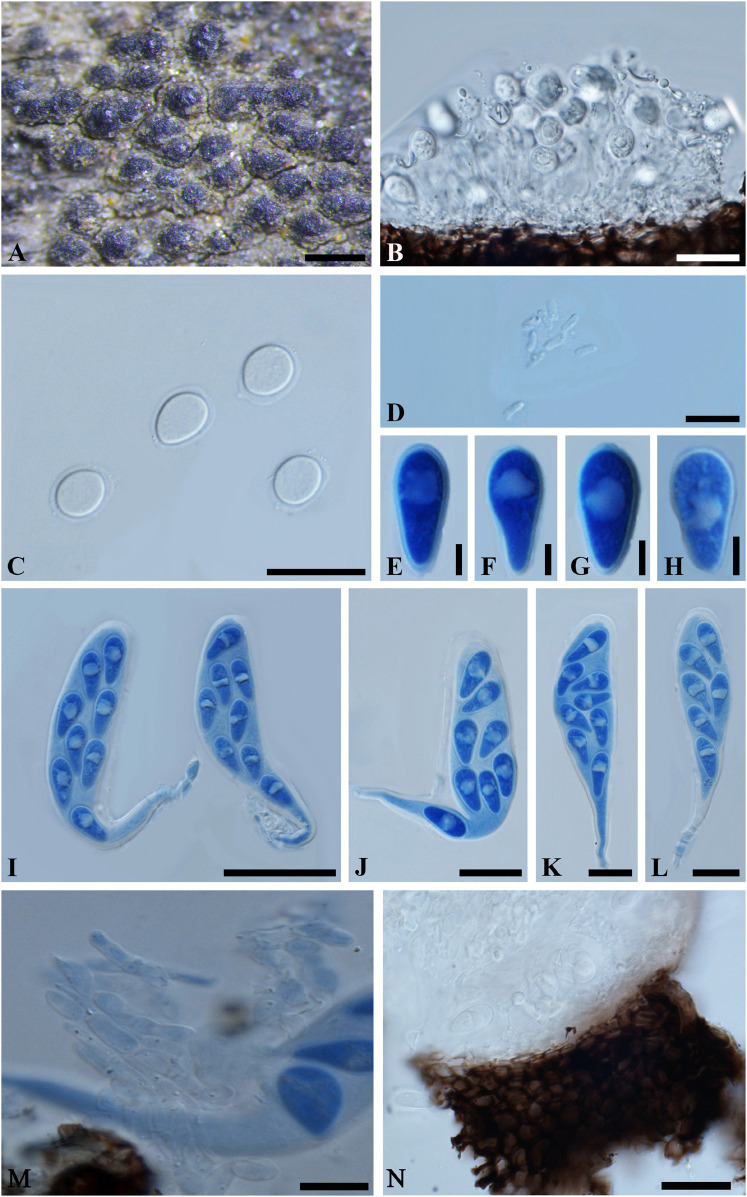
***Neodeightonia mucosa*** (IMI 204341, **type**). **(A)** Aggregated ascomata erumpent through the twig. **(B)** Longitudinal section of a conidioma. **(C)** Conidia surrounded by a thin gelatinous sheath. **(D)** Spermatia. **(E-H)** Ascospores in cotton blue. Note the distinct oil drop in the ascospores. **(I–L)** Broadly clavate asci with a long, narrowed, and twisted pedicel in cotton blue. **(M)** Fragments of cellular pseudoparaphyses. **(N)** Part of peridium. Scale bars: **(A)** = 500 μm, **(B,C,J-L)** = 20 μm, **(D,M,N)** = 10 μm, **(E-H)** = 5 μm, **(I)** = 50 μm.

MycoBank number: 840943; Facesoffungi number: FoF 03579; Figure 4

≡ *Botryosphaeria mucosa* S.J. Kaur, Indian J. Mycol. Plant Path. 25(3): 333 (1996) [1995]

*Ascomata* 170-320 μm diam., erumpent, pseudothecial, scattered, solitary or aggregated, globose with a central ostiole, ¼ to ½ emergent, almost embedded, papillate or not, black. *Peridium* comprising 5–15 layers of *textura angularis*, outer region of dark brown cells, inner region of 2–4 layers of hyaline cells lining the locule. *Asci* bitunicate, broadly clavate, pedicellate, 100-135 × 20–35 μm, 8-spored, with a long, narrowed and twisted pedicel, forming between pseudoparaphyses. *Pseudoparaphyses* filiform, cellular, septate, 3–5 μm wide. *Ascospores* hyaline, aseptate, thin-walled, obovoid to obpyriform, with a distinct oil drop, biseriate in the ascus, 17–20 × 8–10 μm (−x = 18.8 × 8.9 μm, *n* = 20), L/W = 2.1. *Conidiomata* stromatic, pycnidial, solitary or aggregated, morphologically indistinguishable from the ascomata, walls composed of dark brown, thick-walled *textura angularis*, becoming thin-walled and hyaline toward the inner layer. Ostioles single, central, papillate or not. *Paraphyses*, hyaline, septate, 2–3 μm wide. *Conidiophores* reduced to conidiogenous cells. *Conidiogenous cells* hyaline, 8–9(–11) × (1–)2–3 μm. *Conidia* hyaline, aseptate, moderately thick-walled, ovoid or broadly ellipsoidal, surrounded by mucilaginous sheath, 1 μm thick, (7–)9–14 × 6–10(–12) μm (−x = 11.5 × 8.6 μm, *n* = 20), L/W = 1.3. *Spermatia* unicellular, hyaline, allantoid to rod-shaped, 4-6 × 1-2 μm.

Specimen examined – INDIA, Rajasthan, on dead bamboo wood, 25 May 1976 (K 204341, **holotype**).

Notes – The bambusicolous lifestyle, aggregated ascostroma, broadly clavate asci with long pedicel, hyaline, aseptate, thin-walled, obovoid to obpyriform ascospores and its hyaline, aseptate, moderately thick-walled, ovoid or broadly ellipsoidal conidia surrounded by mucilaginous sheath suggest *Neodeightonia* being an appropriate genus for this species. *Neodeightonia* was introduced by Booth (Punithalingam, 1969), and species are typically associated with monocotyledonous plants and especially bamboo (Punithalingam, 1969; Phillips et al., 2008, 2013; Liu et al., 2012; Adamčík et al., 2015; Dai et al., 2017). Morphologically, *Neodeightonia mucosa* is most similar to *Neodeightonia microspora*, while the smaller ascospores (10–12 × 4.5–6 μm) of *N*. *microspora*, can be readily distinguished from those of *N*. *mucosa* (Dai et al., 2017).

So far, six species have been assigned in *Neodeightonia*, namely *Neodeightonia licuriensis*, *N*. *microspora*, *N*. *mucosa*, *Neodeightonia palmicola*, *Neodeightonia phoenicum*, and *Neodeightonia subglobosa*. Of these, *N*. *palmicola* and *N*. *phoenicum* have been reported on palms (Phillips et al., 2008, 2013; Liu et al., 2012), *N*. *subglobosa*, *N. microspora*, and *N*. *mucosa* are reported as bambusicolous (Dai et al., 2017; this study), and *N*. *licuriensis* has been reported on *Syagrus coronata* (Adamčík et al., 2015). *Neodeightonia subglobosa* has also been reported causing keratomycosis in a human eyes (Phillips et al., 2008).

***Neofusicoccum cruenta*** (Petr.) Y.P. Zhou, Y. Zhang ter., comb. nov.

**FIGURE 5:**
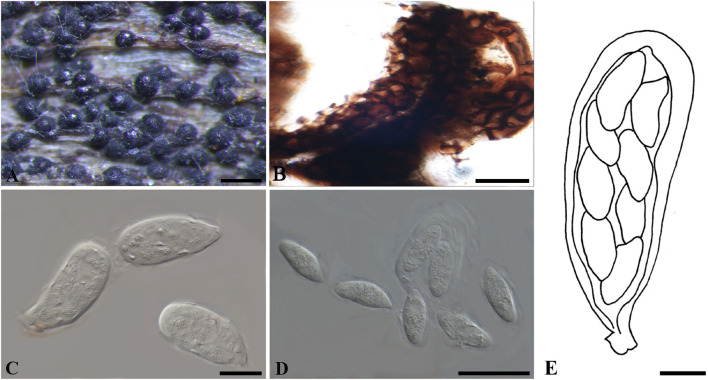
***Neofusicoccum cruenta*** (W 10992, **holotype**). **(A)** Ascomata erumpent through a leaf surface. **(B)** Part of the peridium. **(C,E)** Asci. **(D)** Ascospores. Scale bars: **(A)** = 200 μm, **(B,E)** = 10 μm, **(C,D)** = 20 μm.

MycoBank number: 840944; Facesoffungi number: FoF 03578; Figure 5

≡ *Melanops cruenta* Petr., Annls mycol. 25(3/4): 226 (1927)

*Ascomata* erumpent, 70–140 μm diam., pseudothecial, solitary or gregarious, globose with a central ostiole, ¼ to ½ emergent, rarely embedded, black. *Peridium* composed of 6–10 layers of *textura angularis*, outer region of dark brown cells, inner region of 1–3 layers of hyaline cells lining the locule. *Asci* bitunicate, clavate, 38–65 × 17–22 μm. *Pseudoparaphyses* not observed. *Ascospores* hyaline, fusoid to ellipsoid, sometimes with tapered ends, bi- to triseriate, 13–20 × 5–9 μm (−x = 16.9 × 7 μm, *n* = 20), L/W = 2.4.

Specimen examined – CZECHIA, Prerov, on leaves of *Polygonatum officinale* (*Liliaceae*), April 1926, F. Petrak (W 1978-0010992/24018, **holotype**).

Notes – Only the sexual morph was observed on the type material, the morphology of which is consistent with members of *Botryosphaeriaceae* in having gregarious ascomata, broadly clavate asci and hyaline, aseptate ascospores as well as lacking pseudoparaphyses. Only LSU sequence was obtained for the type material of *Melanops cruenta* in this study, and a few other species of *Neofusicoccum* have LSU sequences available from GenBank as well. The phylogenetic analysis based on these LSU sequences suggested that *M*. *cruenta* resides in *Neofusicoccum*, being sibling to other species in the genus (Supplementary Figure 6). Thus, we have assigned *M*. *cruenta* to *Neofusicoccum* as a new combination, *N*. *cruenta*.

***Neofusicoccum hamamelidis*** (Rehm) Y.P. Zhou, Y. Zhang ter., comb. nov.

**FIGURE 6:**
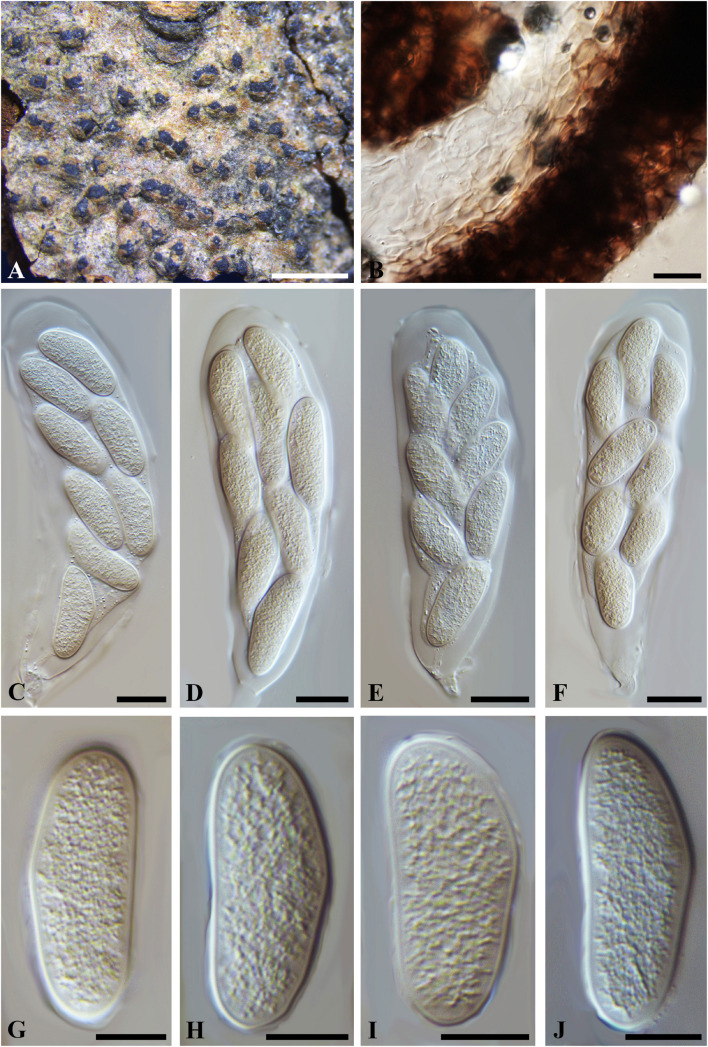
***Neofusicoccum hamamelidis*** (W 29850, **type?**). **(A)** Ascomata erumpent through a twig epidermis. **(B)** Longitudinal section of a peridium showing the cells of *textura angularis*. **(C-F)** Broadly clavate, 8-spored asci with ascospores inside. **(G–J)**. Hyaline, aseptate ascospores. Scale bars: **(A)** = 1 mm, **(B-F)** = 20 μm, **(G-J)** = 10 μm.

MycoBank number: 840945; Facesoffungi number: FoF 03577; Figure 6

≡ *Botryosphaeria hamamelidis* Rehm, Annls mycol. 11(2): 168 (1913)
= *Physalospora laricina* Sawada, Bull. Gov. Forest Exp. Stn 46: 126 (1950)
= *Guignardia laricina* (Sawada) W. Yamam. and Kaz. Itô, Sci. Rep. Hyogo Univ. Agric. 5(1): 9 (1961)
= *Botryosphaeria laricina* (Sawada) Y.Z. Shang, Acta Mycol. Sin. 6(4): 249 (1987)

*Ascomata* erumpent, 170–400 μm diam., pseudothecial, scattered, solitary or aggregated, globose with a central ostiole, ¼ to ½ emergent, almost embedded, black. *Peridium* comprising 5–15 layers of *textura angularis*, outer region of dark brown cells, inner region of 4–6 layers of hyaline cells lining the locule. *Asci* bitunicate, clavate, 110–150 × 30–45 μm, 8-spored, forming among pseudoparaphyses. *Pseudoparaphyses* filiform, septate, constricted at the septa, rarely branched, 2–5 μm broad. *Ascospores* hyaline, thin-walled, ellipsoidal to ovoid, usually broadest in the middle, smooth, contents granular, biseriate in the ascus, 33–39(–48) × 13–16 μm (−x = 36.5 × 14.9 μm, *n* = 20), L/W = 2.4 (some data referred to Kobayashi, 1962).

Specimen examined – CANADA, near London, Ontario, on dead twigs of *Hamamelidis virginiana* (*Hamamelidaceae*), 18 May 1912, J. Dearness (W 07238/29850, **type?**). CHINA, Heilongjiang Province, Tieling, Langxiang, on twigs of *Larix gmelinii* (Rupr.) Kuzen. (*Pinaceae*), 10 July 2015, W. He (HMAS 246968, HMAS 246969).

Notes – The two isolates of *Neofusicoccum hamamelidis* (CGMCC3.18002/CGMCC3.18003) included in the phylogram (Supplementary Figure 5) were obtained from twigs of larch (*Larix gmelinii*) with shoot blight in Heilongjiang Province in China, which had been named as *Physalospora laricina* (on *Larix kaempferi*, Japan, Sawada, 1950), and subsequently combined to *Guignardia* as *G. laricina* (Sawada) W. Yamam. & Kaz. Itô and *Botryosphaeria* and *B. laricina* (Sawada) Y.Z. Shang (Yamamoto, 1961; Shang, 1987). Morphologically, “*Botryosphaeria hamamelidis*” and *P*. *laricina* were almost indistinguishable, but their ascospore broadness (9–12 vs. 13–16 μm, data from HMAS 246968 and HMAS 246969), which was insufficient to split them into two species. The phylogeny based on ITS, *tef1-a*, *tub2* and LSU also supported the conspecific status of *B*. *hamamelidis* and *P*. *laricina* (Supplementary Figures 5, 6). *Botryosphaeria hamamelidis*, as an earlier epithet, had priority over *Physalospora laricina*. Thus, *Physalospora laricina* was reduced to a synonym of *B. hamamelidis*, which was assigned to *Neofusicoccum* as *N*. *hamamelidis* herein.

#### Species Tentatively Included in *Botryosphaeria sensu stricto*

**FIGURE 7:**
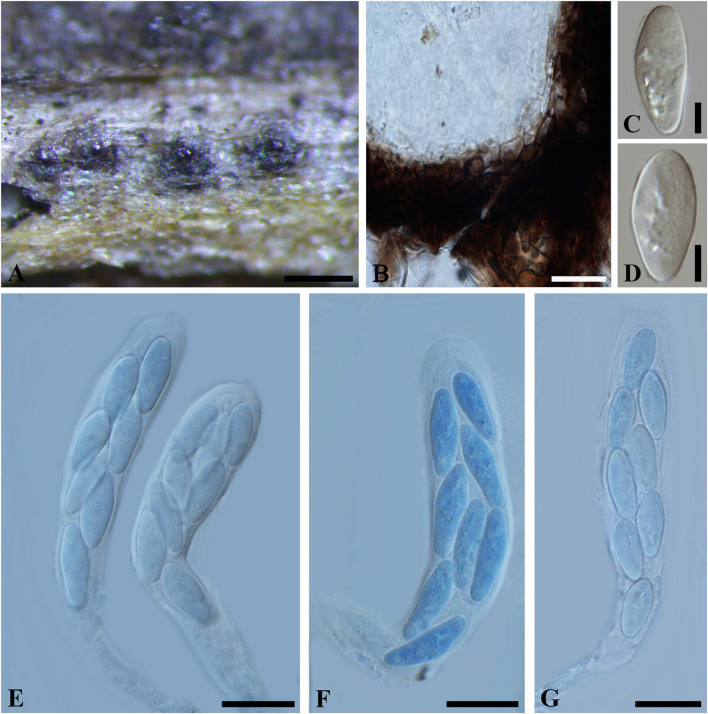
***Botryosphaeria abuensis*** (IMI 192142, **type**). **(A)** Ascostroma erumpent through a twig. **(B)** Part of the peridium. **(C,D)** Released, hyaline ascospores. **(E-G)** Broadly clavate asci in cotton blue. Scale bars: **(A)** = 200 μm, **(E-G)** = 20 μm, **(B-D)** = 5 μm.

***Botryosphaeria abuensis*** S.J. Kaur, 1996, Indian J. Mycol. Plant Path. 25(3): 334 (1996) [1995]. Figure 7

*Ascomata* 120-300 μm diam., pseudothecial, erumpent, scattered, solitary, globose with a central ostiole, ¼ to ½ emergent, rarely embedded, papillate or not, black. *Peridium* comprising 5–15 layers of *textura angularis*, outer region of dark brown cells, inner region of 2–4 layers of hyaline cells lining the locule. *Asci* bitunicate, clavate, pedicellate, up to 20 μm, 8-spored, 80-135 × 18–30 μm, forming among pseudoparaphyses. *Pseudoparaphyses* filiform, cellular, septate, 2–5 μm broad. *Ascospores* oblong to narrowly fusoid, broadest in the upper third, biseriate to triseriate in the ascus, hyaline, smooth, sometimes granular to guttulate, aseptate, (17–)20–30(–32) × 6–11 μm (−x = 23.4 × 8.4 μm, *n* = 30), L/W = 2.8.

Specimen examined – INDIA, Rajasthan, on dead twigs of *Lantana camara* (*Verbenaceae*), 4 March 1975 (K 192142, **type**).

Notes – The solitary or scattered ascomata, clavate asci, hyaline, aseptate, oblong to narrowly fusiform ascospores fit *Botryosphaeriales*. Taxonomic status of this species, however, could not be determined due to a lack of morphological features of the asexual morph and DNA sequence data. Thus *B*. *abuensis* is tentatively kept in *Botryosphaeria sensu stricto* herein.

**FIGURE 8:**
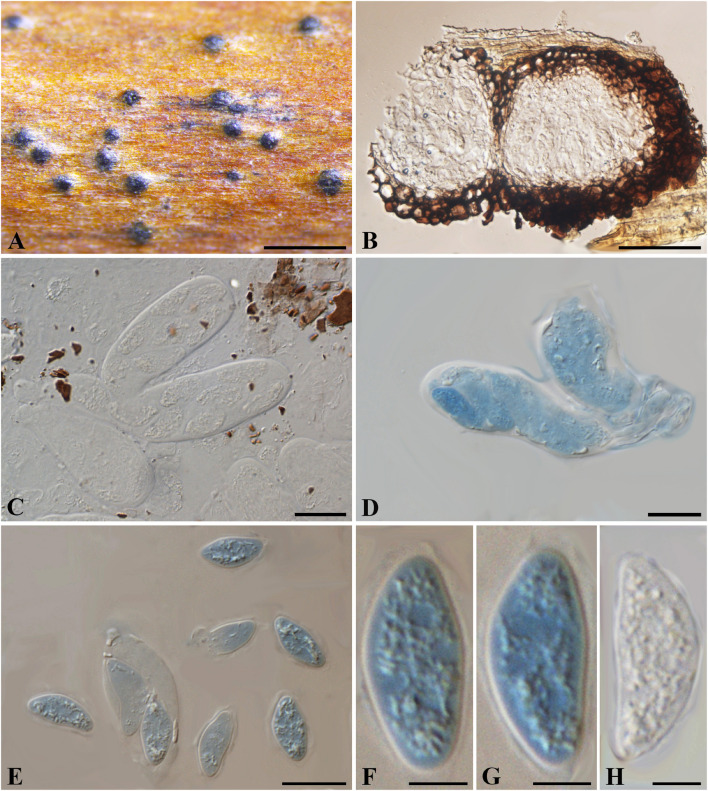
***Botryosphaeria aesculi*** (NYS f93, **holotype**). **(A)** Ascomata erumpent through the bark of host twig. **(B)** Longitudinal section of ascomata. **(C,D)** Squash showing asci (**C** in water, **D** in cotton blue). **(E-H)** Released ascospores (**E-G** in cotton blue, **H** in water). Scale bars: **(A)** = 500 μm, **(B)** = 50 μm, **(C-E)** = 20 μm, **(F-H)** = 5 μm.

***Botryosphaeria aesculi*** (Peck) M.E. Barr, Contr. Univ. Mich. Herb. 2: 561 (1972). Figure 8

≡ *Laestadia aesculi* Peck, Rep. (Annual) Trustees State Mus. Nat. Hist., New York 39: 51 (1887) [1886]

*Ascomata* erumpent, 80-200 μm diam., black, immersed to semi-immersed in the host, becoming erumpent, scattered, sometimes in small groups of 2 locules, globose with a central ostiole, papillate or not. *Peridium* comprising 4–8 layers of *textura angularis*, outer region of dark brown cells, inner region of 1–2 layers of hyaline cells lining the locule. *Pseudoparaphyses* not observed. *Asci* bitunicate, subclavate to clavate, 8-spored, 46-66 × 12–22 μm. *Ascospores* hyaline, ellipsoidal-fusiform or fusiform, broadest in the upper third, irregularly biseriate in the ascus, 16–20(–23) × 6–11 μm (−x = 19 × 8.9 μm, *n* = 20), L/W = 2.1.

Specimen examined – United States, Albany, on petioles of *Aesculus hippocastanum* (*Sapindaceae*), May 1885, G. W. Clinton (NYS f93, **holotype**).

Notes – *Botryosphaeria aesculi* was introduced as *Laestadia aesculi*, which was subsequently assigned to *Botryosphaeria* as *B. aesculi* by Barr (1972). Morphologically, the scattered and erumpent ascomata, small-sized and ellipsoidal asci differ from the species of *Botryosphaeria sensu stricto*, while the hyaline, aseptate and ellipsoidal to fusiform ascospores are consistent with taxa in the *Botryosphaeriales*. Herein we tentatively retain it within *Botryosphaeria* with its taxonomical status remaining to be undetermined until further phylogenetic analysis is carried out on verified specimens.

**FIGURE 9:**
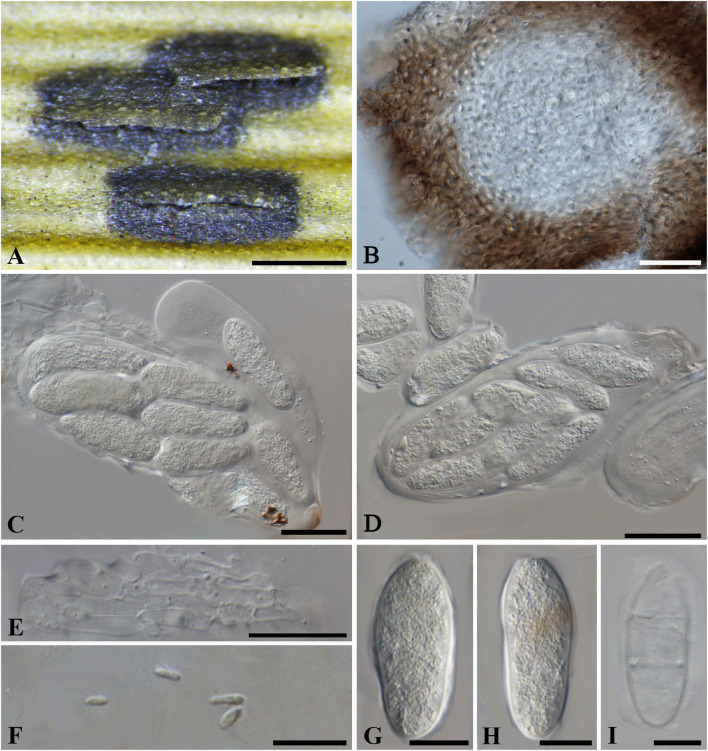
***Botryosphaeria dasylirii*** (NYS f950, **holotype**). **(A)** Ascostroma erumpent through the leaf surface. **(B)** Section of the ascoma showing the peridium. **(C)** Ellipsoidal to narrowly fusiform asci. **(D)** Breaking asci with released ascospores. **(E)** Septate pseudoparaphyses. **(F)** Spermatia. **(G,H)** Hyaline aseptate ascospores with granular content. **(I)** Aged, 2-septate, hyaline ascospores. Scale bars: **(A)** = 500 μm, **(C-E)** = 20 μm, **(B,F-I)** = 10 μm.

***Botryosphaeria dasylirii*** (Peck) Theiss. and Syd., Annls mycol. 13(5/6): 663 (1915). Figure 9

≡ *Dothidea dasylirii* Peck, Bot. Gaz. 7(5): 57 (1882)

*Ascostroma* erumpent through the leave surface, 0.3-1.8 mm diam. *Ascomata* 90-150 μm diam., pseudothecial, usually forming botryose clusters of 3-4 locules, globose with a central ostiole, covered with epidermal leaf tissue, black. *Peridium* comprising 9–12 layers of *textura angularis*, outer region of dark brown cells, inner region of 2–3 layers of hyaline cells lining the locule. *Asci* 8-spored, bitunicate, ellipsoidal to subclavate, 100-115 × 23–40 μm, forming between pseudoparaphyses. *Pseudoparaphyses* filiform, cellular, septate, 3–4 μm wide. *Ascospores* hyaline, ovoid to narrowly ellipsoid with granular content, aseptate, biseriate to triseriate, (24–)26–37(–40) × 10–15 μm (−x = 33 × 14 μm, *n* = 20), L/W = 2.3, sometimes becoming 0–2 septa with aging. *Spermatia* unicellular, hyaline, allantoid to rod-shaped, 4.5-6 × 1.5-2 μm.

Specimen examined – United States, Arizona, on leaves of *Dasylirion* sp. (*Asparagaceae*), May 1881, C. G. Pringle (NYS f950, **holotype**).

Notes – The erumpent botryose ascostroma, cellular pseudoparaphyses, hyaline, aseptate, large-sized ascospores suggest an affiliation in the *Botryosphaeriaceae*, while the ovoid to narrowly ellipsoid ascospores fit both *Botryosphaeria* and *Neofusicoccum*. Since there are no obvious morphological differences between *Neofusicoccum* and *Botryosphaeria*, the generic status of *B*. *dasylirii* remains uncertain until DNA sequence data can be obtained for it. Thus *B. dasylirii* is tentatively kept in *Botryosphaeria sensu stricto* herein until further phylogenetic analysis is carried out on verified specimens.

***Botryosphaeria wisteriae*** (Rehm) Sacc., Syll. fung. (Abellini) 1: 459 (1882) Figure 10

≡ *Thuemenia wisteriae* Rehm, Mycoth. Univ., cent. 10: no. 971 (in sched.) (1878)

**FIGURE 10:**
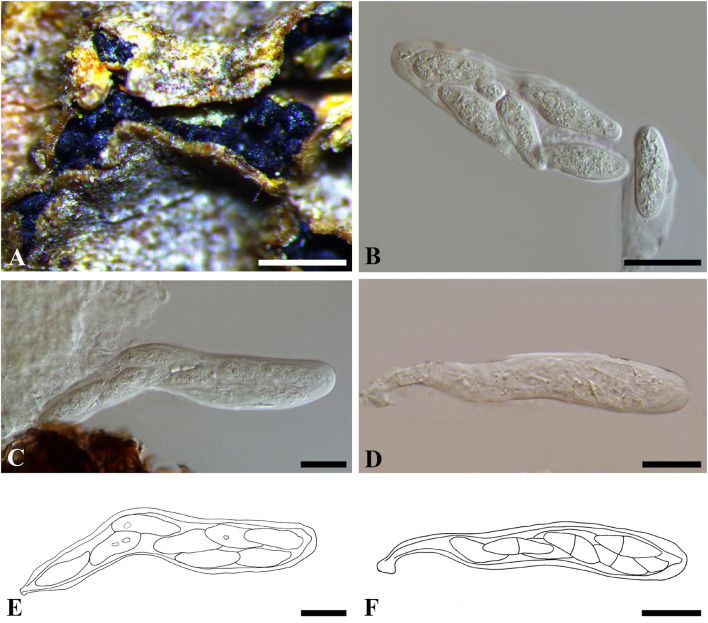
***Botryosphaeria wisteriae*** (MICH 15081, **isotype**). **(A)** Botryose clustered ascomata erumpent through a twig epidermis. **(B)** Broken ascus showing hyaline, narrowly fusiform, ascospores within it. **(C-F)** Clavate ascus with aseptate or separated ascospores. Scale bars: **(A)** = 500 μm, **(B-F)** = 10 μm.

*Ascostroma* erumpent from bark of host, 0.5–1.5 mm diam. *Ascomata* 140-220 μm diam., pseudothecial, botryose clustered, globose with a central ostiole, black. *Peridium* comprising 7–13 layers of *textura angularis*, outer region of dark brown cells, inner region of 2–3 layers of hyaline cells lining the locule. *Asci* 8-spored, bitunicate, clavate with a short pedicel, 70-118 × 17–27 μm, forming between pseudoparaphyses. *Pseudoparaphyses* filiform, cellular, septate, 3–5 μm broad. *Ascospores* hyaline, ellipsoid to fusiform, granular content not sure, partially overlapping to biseriate in ascus, sometimes become 1-septate with aging, (17–)20–26(–30) × 7–10.5 μm (−x = 23.6 × 9.1 μm, *n* = 20), L/W = 2.6. *Spermatia* not observed.

Specimen examined – United States, South Carolina, Aiken, on dead twig of *Wisteria chinensis* (*Fabaceae*), Thuemen (MICH 15081, **isotype**).

Notes – The botryose ascomata, cellular pseudoparaphyses, and aseptate, hyaline ascospores are consistent with members of the *Botryosphaeria sensu stricto*. Thus *B. wisteriae* is tentatively kept in *Botryosphaeria sensu stricto* herein until further phylogenetic analysis is carried out on verified specimens.

#### Taxa Excluded From *Botryosphaeriales*

***Nothophoma ferruginea*** (Fuckel) Y.P. Zhou and Y. Zhang ter., comb. nov.

**FIGURE 11:**
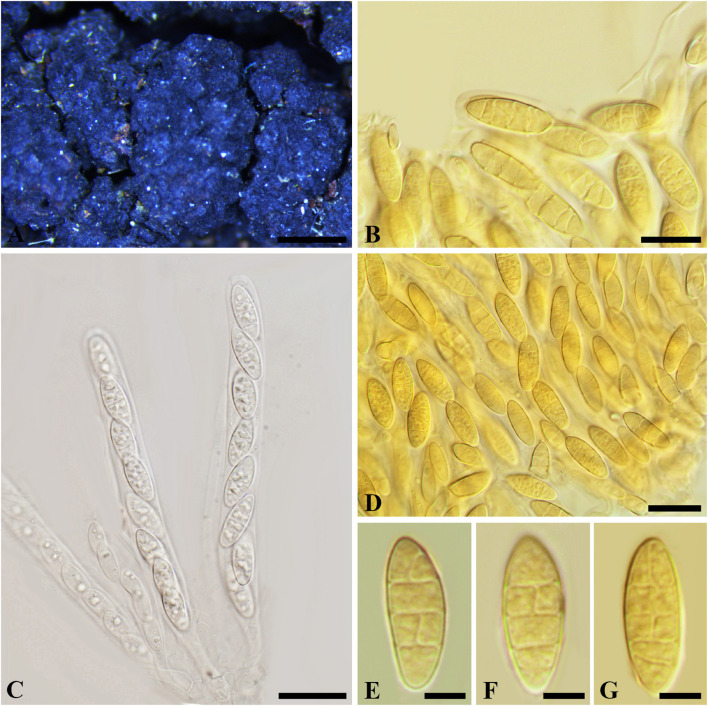
***Nothophoma ferruginea*** (G 00127285, **holotype**). **(A)** Densely gregarious ascomata. **(B,D-G)** Ascospores in Melzer’s reagent. **(C)** Ascus with ascospores in water. Scale bars: **(A)** = 1 mm, **(B)** = 10 μm, **(C,D)** = 20 μm, **(E-G)** = 5 μm.

MycoBank number: 840946; Facesoffungi number: FoF 03576; Figure 11

≡ *Melanops ferruginea* Fuckel, Jb. nassau. Ver. Naturk. 25-26: 96 (1873)
≡ *Botryosphaeria ferruginea* (Fuckel) Sacc., Syll. fung. (Abellini) 1: 465 (1882)

*Ascostroma* immersed to erumpent, 2–8 mm diam. *Ascomata* 220-550 μm diam., densely gregarious, with elongate, rounded, obtuse or acute papilla, black. *Peridium* comprising 12–15 layers of *textura angularis*, outer region of dark brown cells, inner region of 3–5 layers of hyaline cells lining the locule. *Asci* 8-spored, bitunicate, cylindrical with a short, narrowed, twisted, furcate pedicel, 115-145 × 10–13 μm, forming between pseudoparaphyses. *Pseudoparaphyses* filiform, cellular, septate, 3–4 μm wide. *Ascospores* uniseriate to partially overlapping, ellipsoidal to ovoid, muriform, with 3–4 transversal septa and 1–2 longitudinal septa in first, second or third cell(s), hyaline, (14–)17–20 × 6–9 μm (−x = 15.6 × 7.5 μm, *n* = 20), L/W = 2.5.

Specimen examined – SWITZERLAND, Neuchâtel, on the trunks of corruption *Alnus glutinosa* (*Betulaceae*), February 1872, Morthier (G 00127285, **holotype**).

Notes –The bitunicate asci, cellular pseudoparaphyses, the hyaline, broadly ellipsoid ascospores with 3–4 transversal septa and 1–2 longitudinal septa in central cells of *Melanops ferruginea* differ from members of *Botryosphaeriales*. Phylogeny based on ITS and LSU nuDNA sequences indicated that *M*. *ferruginea* closely related to *Nothophoma* Qian Chen and L. Cai (*Didymellaceae, Pleosporales*). Therefore, we assign it to *Nothophoma* as *N. ferruginea*.

**FIGURE 12:**
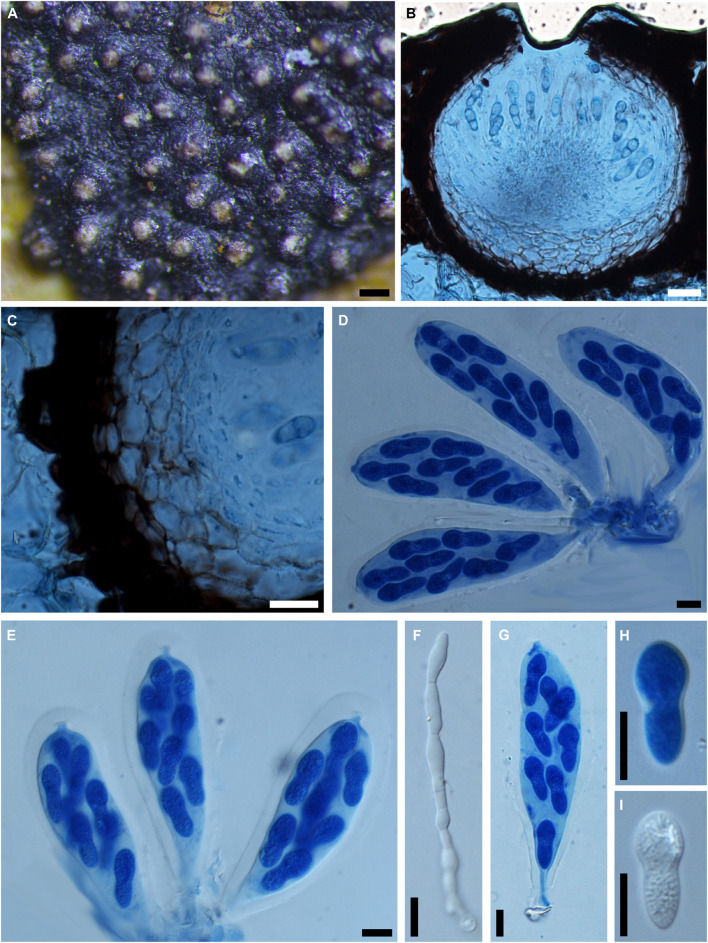
***Phyllachorella micheliae*** (IMI 316002, **holotype**). **(A)** Botryose clusters of ascomata erumpent through the lower side of the leaf. **(B)** Longitudinal section through an ascoma in cotton blue. **(C)** Section of the peridium comprising cells of *textura angularis* in cotton blue. **(D,E,G)** Squash mounts showing broadly clavate asci with wide ocular chamber near the apex and short pedicels at the base in cotton blue. **(F)** Septate pseudoparaphyses in water. **(H,I)** Hyaline, aseptate ascospores in cotton blue **(H)** or in water **(I)**. Scale bars: **(A)** = 100 μm, **(B)** = 20 μm, **(C-I)** = 20 μm.

***Phyllachorella micheliae*** Syd., Annls mycol. 12(5): 489 (1914). Figure 12

= *Botryosphaeria foliicola* (Sivanesan and Nair, 1988)

*Ascomata* 140-250 μm diam., pseudothecial, aggregated forming a large botryose, irregularly rounded, 2–8 mm diam., lower side of the leaves, globose with a central white ostiole, ostiole 20–40 μm broad, ½ to ¾ emergent, rarely embedded, black. *Peridium* thick-walled, comprising 5–10 layers of *textura angularis*, outer region of dark brown cells, inner region of 4–6 layers of hyaline cells lining the locule. *Asci* 8-spored, bitunicate, broadly clavate with a long, narrow pedicel, which is up to 80 μm, with obvious apical chamber, 90-140 × 20–30 μm, forming between pseudoparaphyses. *Pseudoparaphyses* filiform, hyaline, cellular, septate, obviously constricted at the septa, rarely branched, 4–5 μm wide. *Ascospores* hyaline, aseptate, thin-walled, unequally gourd-shaped with upper part broader than the lower part, biseriate to triseriate in the ascus, (16–)18–21(–22) × 8–9(–11) μm (−x = 19.4 × 8.8 μm, *n* = 20), L/W = 2.2.

Specimen examined – INDIA, Kodaikanal, Tamil Nadu, on leaves of *Michelia nilgarica* (*Magnoliaceae*), 10 January 1987, L. N. Nair (K 316002, **holotype**).

Notes – *Botryosphaeria foliicola* was introduced by Sivanesan and Nair (1988) from the leaves of *Michelia nilgarica* in India, which distinguishes it from other species of *Botryosphaeria* by its obovoid and characteristically constricted ascospores. *Phyllachorella micheliae*, the generic type of *Phyllachorella*, was reported from the leaves of the same host in India. The strong morphological similarity of *Botryosphaeria foliicola* and *Phyllachorella micheliae* warranted their conspecific status (Liu et al., 2012). Based on priority, the later name *Botryosphaeria foliicola* is reduced to synonymy with *Phyllachorella micheliae*, which is retained in *Botryosphaeriales genera incertae sedis* (Wijayawardene et al., 2020).

#### Dothideomycetes incertae sedis

***Botryosphaeria gaubae*** Petr., Sydowia 21: 235 (1968). Supplementary Figure 1

*Ascomata* erumpent, 200-360 μm diam. *Ascomata* scattered, solitary, externally black, globose with a central ostiole, ¼ to ½ emergent, embedded in hairy seta on lower side of the leaves. *Peridium* comprising 5–15 layers of *textura angularis*, outer region of dark brown cells, inner region of 3–5 layers of hyaline cells lining the locule. *Asci* bitunicate, cylindrical or broadly cylindrical, pedicellate or not, pedicles up to 20 μm, 8-spored, 140-180 × 25–35 μm, forming among pseudoparaphyses. *Pseudoparaphyses* filiform, narrowly cellular, rarely branched, 2–3 μm broad. *Ascospores* hyaline, aseptate, fusiform to ellipsoid, with tapered ends and appearing spindle-shaped, sometimes slightly narrower at the middle, irregularly biseriate in the ascus, (23–)27–36(–46) × (6–)10–15(–17) μm (−x = 32.2 × 12.6 μm, *n* = 20), L/W = 2.6.

Specimen examined – AUSTRALIA, on leaves of *Grevillea victoriae* (*Proteaceae*), Mt Franklin, *ca*. 4500 ft., 27 January 1953, leg. E. Gauba (W 1992-05937, **holotype**).

Notes – *Botryosphaeria gaubae* was introduced and assigned in *Botryosphaeria sensu lato*, which included *Botryosphaeria*, *Gibberella*, and *Lisea* or even *Melanops* (Petrak, 1967). While the foliicolous habitation, scattered ascomata, cylindrical asci, fusiform to ellipsoid, aseptate ascospores with tapered ends and the presence of filiform pseudoparaphyses differ from these genera. We consequently treat it as a species in the *Dothideomycetes incertae sedis*.

***Laestadia apocyni*** Ellis and Everh., Proc. Acad. nat. Sci. Philad. 42: 230 (1890). Supplementary Figure 2

= *Botryosphaeria apocyni* (Ellis and Everh.) M.E. Barr, Contr. Univ. Mich. Herb. 9(8): 560 (1972)

*Ascomata* 100-215 μm diam., pseudothecial, scattered or clustered, globose with a central ostiole, papillate or not, black. *Peridium* thin, comprising 2–3 layers of *textura angularis*. *Asci* 8-spored, bitunicate, slightly obclavate, lack of pedicel, 47 × 27 μm (only a single complete mature ascus observed). *Pseudoparaphyses* not observed. *Ascospores* hyaline, fusiform with rounded ends, 1-septate, biseriate in the ascus, 14–20 × 4–8 μm (−x = 17.2 × 6.1 μm, *n* = 10), L/W = 2.8.

Specimen examined – CANADA, Ontario, Middlesex: London, on dead stems of *Apocynum* sp. (*Apocynaceae*), J. Dearness (MICH 14281, **isotype**).

Notes – The small-sized ascomata, bitunicate, slightly obclavate asci, lack of a pedicel, hyaline, 1-septate ascospores disagree with *Botryosphaeria sensu stricto*. *Laestadia apocyni* had been assigned to *Guignardia* as *G. apocyni*, while its 1-septate ascospores disagree with the non-septate ascospores of *Guignardia* (Vasyagina et al., 1987; Barr, 1993). Thus, its taxonomic status cannot be determined yet, and tentatively assigned in *Dothideomycetes incertae sedis*.

***Sphaeria smilacinina*** Peck, Ann. Rep. N.Y. St. Mus. nat. Hist. 29: 62 (1878) [1876] Supplementary Figure 3

= *Botryosphaeria smilacinina* (Peck) M.E. Barr, Contr. Univ. Mich. Herb. 9(8): 560 (1972)
= *Discochora smilacinina* (Peck) Bissett, Can. J. Bot. 64(8): 1721 (1986)

*Ascomata* 150-280 μm diam., pseudothecial, solitary or scattered, globose with a central ostiole, immersed or ¼ to ½ emergent, black. *Peridium* comprising 6–12 layers of *textura angularis*, outer region of dark brown cells, inner region of 2–4 layers of hyaline cells lining the locule. *Asci* bitunicate, broadly clavate to broadly cylindrical, 100-150 × 23–36 μm, forming among pseudoparaphyses. *Pseudoparaphyses* filiform, cellular, septate, 3–5 μm broad. *Ascospores* fusiform to narrowly fusiform, 1-septate, obviously constricted in the middle, biseriate in the ascus, 26–32 × 8–11 μm (−x = 29.6 × 9.4 μm, *n* = 10), L/W = 3.2.

Specimen examined – United States, Albany, on dead stems of *Smilacina stellate* (*Liliaceae*), Charles H. Peck (NYS f2818, **holotype**).

Notes – *Sphaeria smilacinina* has been assigned to *Discochora* as *D. smilacinina* (*Phyllostictaceae*, *Botryosphaeriales*) (Bissett, 1986). The 1-septate ascospore of *S*. *smilacinina*, however, disagree with the non-septate ascospores of *Discochora*. The immersed and scattered, ostiolate pseudothecia, clavate to broadly cylindrical bitunicate asci, 1-septate, constricted, hyaline ascospores suggest that this taxon probably resides in the *Didymellaceae* (*Pleosporales*), while its taxonomic status cannot be determined until further phylogenetic analysis is carried out on verified specimens (Zhang et al., 2012; Chen et al., 2015). Thus, we tentatively keep this species in *Dothideomycetes incertae sedis*.

## Discussion

*Botryosphaeria sensu lato* was described mainly on the basis of morphological characters of its sexual morph and host associations, which led to 286 epithets being assigned to the genus (Index Fungorum, 22/08/2021)^3^. *Botryosphaeria sensu lato* was characterized based on its pseudothecia, ostiolate, often multiloculate ascostroma, cellular pseudoparaphyses, bitunicate, uni- to biseriate, 8-spored asci with or without pedicels, aseptate, ovoid to fusoid to ellipsoid ascospores which may become brown and 1–2-septate with age. The genus has been connected with numerous asexual genera including *Diplodia*, *Dothiorella*, *Lasiodiplodia*, *Macrophoma*, *Sphaeropsis*, and *Fusicoccum* (Sivanesan, 1984; Barr, 1987; Denman et al., 2000). Based on the phylogenetic analysis of 28S rDNA sequences, Crous et al. (2006) recognized ten clades in the *Botryosphaeriaceae*, and noted that the morphology of the conidial morphs was more informative in generic circumscription. Thus far, *Botryosphaeria sensu lato* was reported being highly polyphyletic with only eight species being treated in *Botryosphaeria sensu stricto* (Crous et al., 2006; Phillips et al., 2008, 2013; Slippers et al., 2014; Ariyawansa et al., 2016; Zhou et al., 2016, 2017; Hattori et al., 2021; Zhang et al., 2021).

Four of the 17 taxa of *Botryosphaeria sensu lato* considered in the present study have been confirmed as members of *Botryosphaeria sensu stricto*, which include *B*. *berengeriana* var. *acerina*, *B*. *aterrima*, *B*. *berengeriana var*. *weigelae*, and *B*. *mirabile* with both *B*. *berengeriana* var. *acerina* and *B*. *berengeriana* var. *weigelae* reduced to synonyms of *B. dothidea*. Two other species of *Botryosphaeria sensu lato* were assigned in *Neofusicoccum*, *viz*., *N*. *cruenta* and *N*. *hamamelidis*. *Neofusicoccum* was separated from *Botryosphaeria* and introduced as a new genus based on combined multigene phylogenetic analysis and subtle morphological differences, i.e., pycnidial paraphyses only exist in *Botryosphaeria* (*Fusicoccum*), which have never been reported in any *Neofusicoccum* species (Crous et al., 2006; Phillips et al., 2013). Because of their morphological similarities, it is possible or even probable that some other species of *Botryosphaeria sensu lato* may actually more appropriately reside in *Neofusicoccum*.

Based on the phylogenetic analyses of combined ITS, LSU, *tef1-a* and *tub2* loci, *B*. *laricina* and *N. hamamelidis* (= *B*. *hamamelidis*) form a conspecific clade. The morphological characteristics of their sexual morphs also support their conspecific status (see comments above). *Neofusicoccum hamamelidis* was originally reported from the dead twigs of *Hamamelidis virginiana* in Canada, while *B. laricina* causes shoot blight of larch, which is one of the most important quarantine diseases in China (Liu et al., 2009). The conspecific status of *N. hamamelidis* and *B*. *laricina* supports its broad host range and wide distribution, and these will help in making practical quarantine rules, as a comprehensive knowledge as well as accurate identification of pathogens are extremely important when formulating quarantine regulations (Kumar et al., 2008).

The sexual morph of *Botryosphaeria sensu lato* is morphologically conserved (for example, in the size of the asci and ascospores), while the morphology of asexual morph and host association varies considerably more, which contributes to a natural classification of this group of fungi (Shoemaker, 1964; Pennycook and Samuels, 1985; Phillips et al., 2013; Slippers et al., 2014). For example, *Botryosphaeria mucosa* was assigned to *Neodeightonia* (as *N*. *mucosa*, *Botryosphaeriaceae*) based on its bambusicolous host association, aggregated ascostroma, shape of asci, ascospore shape and septation, as well as conidial morphology. Based on the morphological characteristics or DNA sequences comparisons, many specimens considered in this study should be excluded from *Botryosphaeriales*. For example, *B*. *ferruginea* in *Nothophoma* as *N*. *ferruginea* (*Pleosporales*), *Botryosphaeria apocyni* (Basionym: *Laestadia apocyni*), *B*. *gaubae* and *B*. *smilacinina* (Basionym: *Sphaeria smilacinina*) in *Dothideomycetes incertae sedis*. Thus, a polyphasic taxonomic approach should be applied in type studies of *Botryosphaeria sensu lato*, including the use of host association, morphological characteristics of both sexual and asexual morphs, geographical distribution, DNA sequences as well as epitypification where possible.

Based on both morphological characters and results of *nu*DNA sequence analysis, *Botryosphaeria sensu stricto* now includes ten species, namely *B. agaves, B. aterrima, B. corticis, B. dothidea, B. fabicerciana, B. kuwatsukai, B. mirabile, B. qingyuanensis, B. ramosa* and *B. scharifii*, of which *B*. *fabicerciana* and *B. qingyuanensis* have previously been reported from China. The current study shows that further studies are necessary on other type specimens of *Botryosphaeria sensu lato* in order to clarify their taxonomic status. Fresh collections are also needed to facilitate their epitypification.

In summary, *Botryosphaeria sensu lato* is highly polyphyletic, and species belong to various genera or families of *Botryosphaeriales* or even other orders within *Dothideomycetes*. Studying the type material of *Botryosphaeria sensu lato* helps to understand the circumscription of genera or families within *Botryosphaeriales*. Redescribing and obtaining DNA sequences of the type specimens makes it possible to epitypify those species and clarify their taxonomic status (Zhang et al., 2008). Of the 286 epithets within *Botryosphaeria sensu lato*, less than 20% have DNA sequences available from the type materials (Denman et al., 1999; Smith et al., 2001; Slippers et al., 2004; Ariyawansa et al., 2016; Zhang et al., 2021). Thus, further study is required to obtain a more natural classification for species presently accommodated in *Botryosphaeria sensu lato*.

## Data Availability Statement

The data presented in the study are deposited in the TreeBASE and GenBank repository, accession numbers are S21054 for *Botryosphaeria*, S21059 and S21050 for *Neofusicoccum*, GenBank accession are listed in Supplementary Table 1.

## Author Contributions

YZ designed the experiments. YZ and YPZ prepared the samples, conducted the molecular experiments, and analyzed the data. WS, LZ, DP-Z, PC, BS, and YD revised the manuscript. All authors contributed to the article and approved the submitted version.

## Conflict of Interest

The authors declare that the research was conducted in the absence of any commercial or financial relationships that could be construed as a potential conflict of interest.

## Publisher’s Note

All claims expressed in this article are solely those of the authors and do not necessarily represent those of their affiliated organizations, or those of the publisher, the editors and the reviewers. Any product that may be evaluated in this article, or claim that may be made by its manufacturer, is not guaranteed or endorsed by the publisher.

## Footnotes

1
http://www.mycobank.org

2
https://www.ncbi.nlm.nih.gov/genbank/

3
http://www.indexfungorum.org

## Supplementary Material

Supplementary Figure 1 ***Botryosphaeria gaubae*** (W 1992-05937, **holotype**). **(A,B)** Ascomata erumpent through the lower side of the leaf. **(C)** Squash showing cylindrical or broadly cylindrical asci in cotton blue. **(D)** Part of the peridium. **(E)** Septate pseudoparaphyses in cotton blue. **(F-H)** Aseptate, fusiform to ellipsoid ascospores in cotton blue. Scale bars: **(A)** = 1 mm, **(B)** = 200 μm, **(C)** = 50 μm, **(E)** = 20 μm, **(D,F-H)** = 10 μm.

Supplementary Figure 2 ***Laestadia apocyni*** (MICH 14281, **isotype**). **(A)** Ascomata erumpent through a piece of twig epidermis. **(B)** Released, hyaline, 1-septate ascospores. **(C)** Ascus in water. **(D)** Line drawing of ascus in water. Scale bars: **(A)** = 200 μm, **(B-D)** = 20 μm.

Supplementary Figure 3 ***Sphaeria smilacinina*** (NYS f2818, **holotype**). **(A)** Ascomata erumpent through the twig epidermis. **(B,C)** Immature asci. **(D)** Released ascospores. **(E)** Line drawing of broadly clavate ascus. Scale bars: **(A)** = 500 μm, **(B-D)** = 20 μm, **(E)** = 40 μm.

Supplementary Figure 4 One of the most parsimonious trees obtained from combined ITS, LSU, *tub2*, and *tef1-a* sequence data of *Botryosphaeria* spp. Outgroup taxa are *Neofusicoccum luteum* and *Neofusicoccum parvum*. Maximum parsimony (MP) support values above 70% and Bayesian posterior probabilities (PP) support above 80% are shown with MP bootstrap followed by Bayesian PP (MP/PP) values at the nodes. The species characterized in this study are in boldface.

Supplementary Figure 5 One of the most parsimonious trees obtained based on combined ITS, *tef1-α*, and *tub2* sequence data of *Neofusicoccum* spp. Outgroup taxon are *Botryosphaeria dothidea* and *B. corticis*. Maximum parsimony (MP) support values above 60% and Bayesian posterior probabilities (PP) support above 80% are shown with MP/PP, values at the nodes. The species characterized in this study are in boldface.

Supplementary Figure 6 One of the most parsimonious trees obtained from LSU sequence dataset of *Neofusicoccum* spp. Outgroup taxa are *Botryosphaeria corticis* and *B*. *dothidea*. Maximum parsimony (MP) support values above 70% and Bayesian posterior probabilities (PP) support above 80% are shown with MP bootstrap followed by Bayesian PP (MP/PP) values at the nodes. The species characterized in this study are in boldface.

Supplementary Figure 7 One of the most parsimonious trees obtained from ITS and LSU sequence dataset of *Nothophoma* spp. Outgroup taxa is *Didymella calidophila*. Maximum likelihood (ML) support values above 50%, Maximum parsimony (MP) support values above 50%, and Bayesian posterior probabilities (PP) support above 95% are shown with ML and MP bootstrap followed by Bayesian PP (MP/PP/ML) values at the nodes. The species characterized in this study are in boldface.

Supplementary Table 1 Species, specimens and GenBank accession numbers of sequences used in this study (newly generated sequences are indicated in bold).
