# Supplementary figures and images for: Toward a Natural Classification of Botryosphaeriaceae: A Study of the Type Specimens of Botryosphaeria sensu lato
Source: Front Microbiol. 2021 Nov 3;12:737541. doi: 10.3389/fmicb.2021.737541 (PMC8595605; doi:10.3389/fmicb.2021.737541)

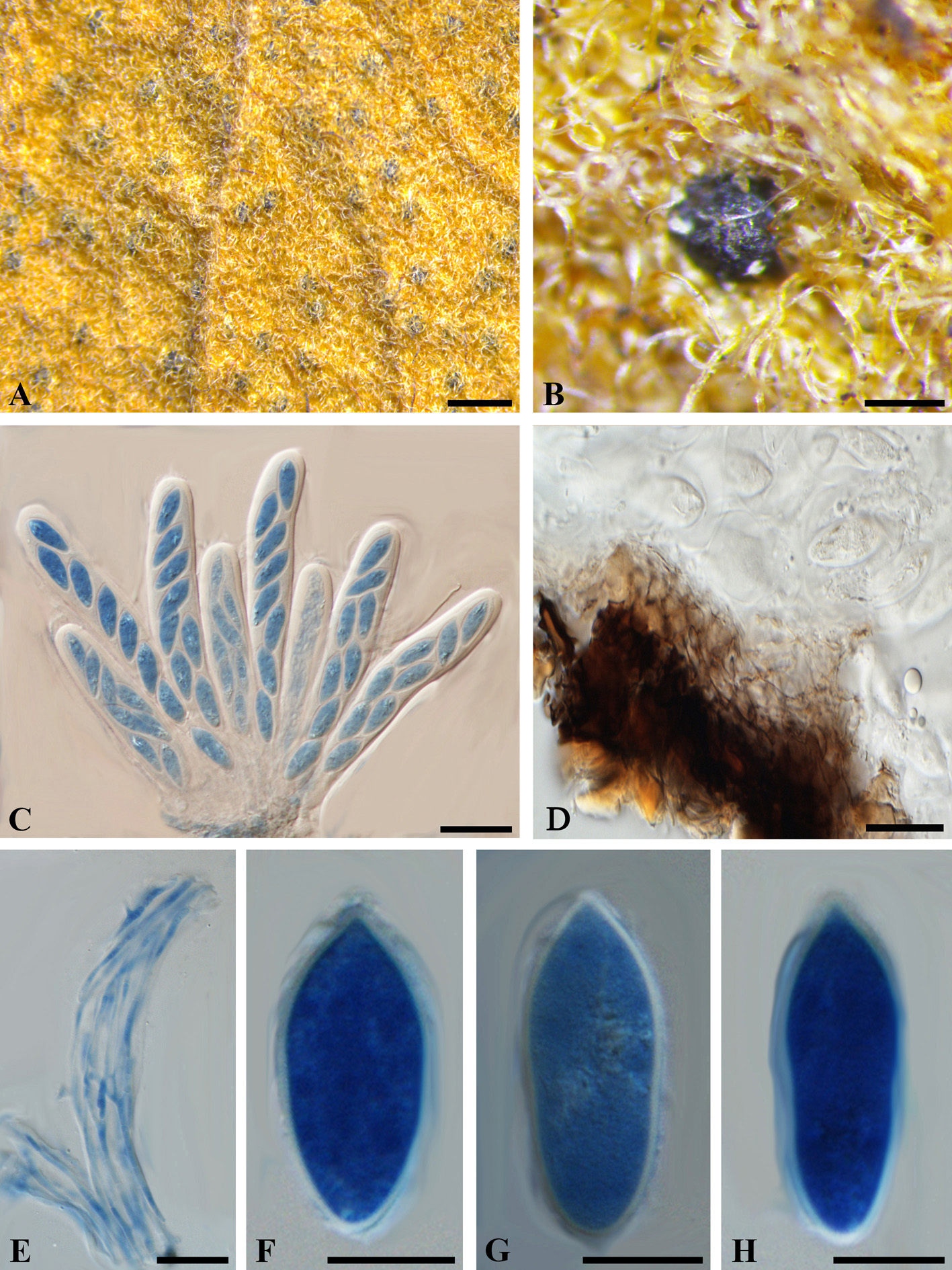

Supplement: Supplementary Figure 1 — Botryosphaeria gaubae (W 1992-05937, holotype). (A,B) Ascomata erumpent through the lower side of the leaf. (C) Squash showing cylindrical or broadly cylindrical asci in cotton blue. (D) Part of the peridium. (E) Septate pseudoparaphyses in cotton blue. (F-H) Aseptate, fusiform to ellipsoid ascospores in cotton blue. Scale bars: (A) = 1 mm, (B) = 200 μm, (C) = 50 μm, (E) = 20 μm, (D,F-H) = 10 μm. [file Image_1.JPEG]

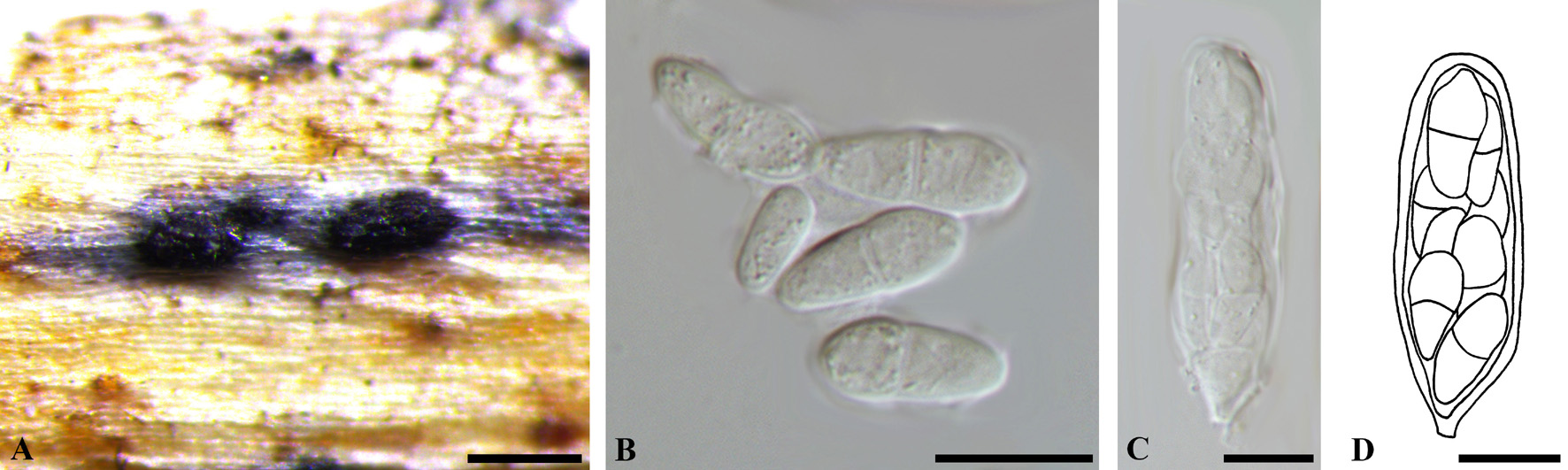

Supplement: Supplementary Figure 2 — Laestadia apocyni (MICH 14281, isotype). (A) Ascomata erumpent through a piece of twig epidermis. (B) Released, hyaline, 1-septate ascospores. (C) Ascus in water. (D) Line drawing of ascus in water. Scale bars: (A) = 200 μm, (B-D) = 20 μm. [file Image_2.JPEG]

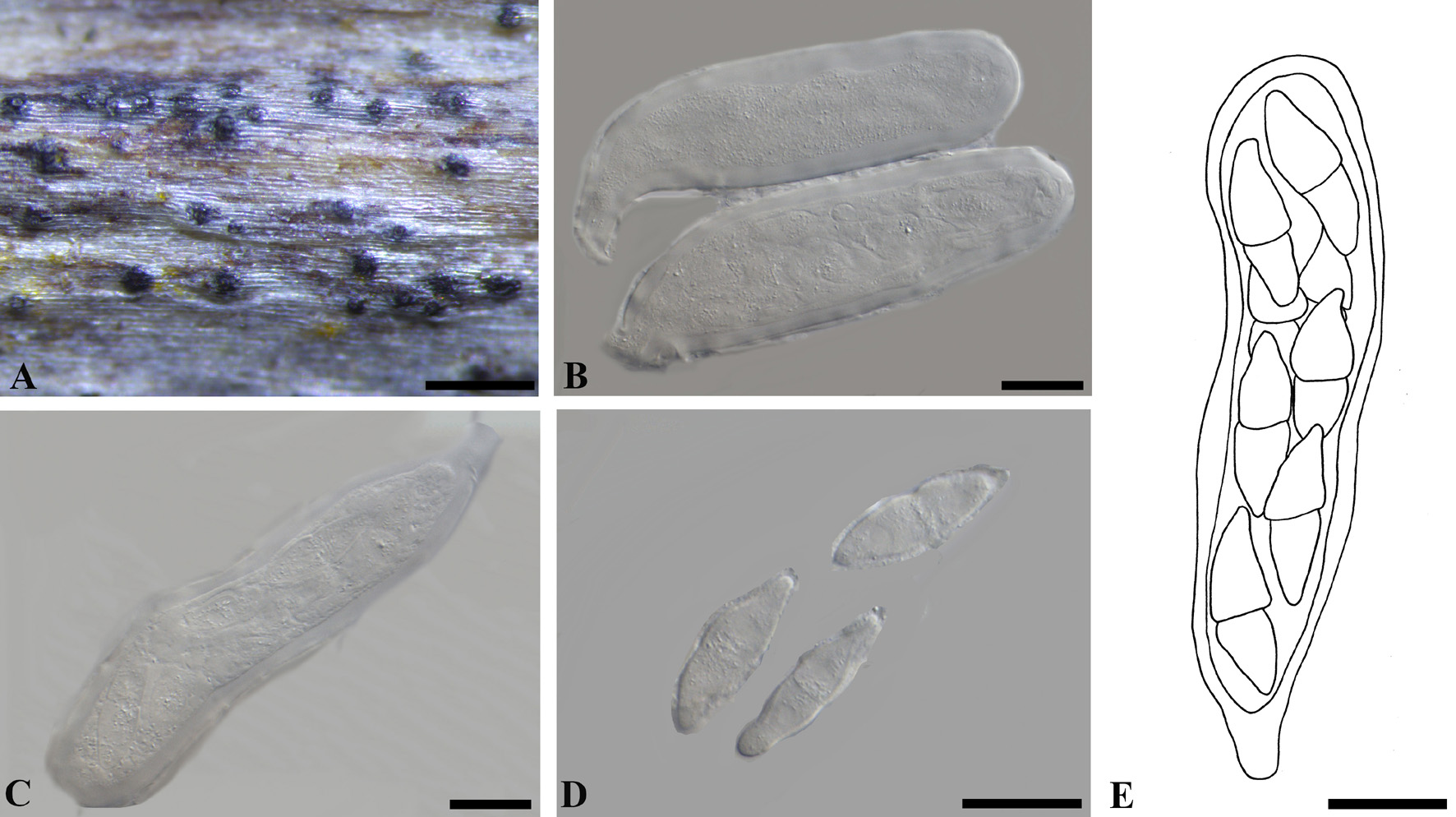

Supplement: Supplementary Figure 3 — Sphaeria smilacinina (NYS f2818, holotype). (A) Ascomata erumpent through the twig epidermis. (B,C) Immature asci. (D) Released ascospores. (E) Line drawing of broadly clavate ascus. Scale bars: (A) = 500 μm, (B-D) = 20 μm, (E) = 40 μm. [file Image_3.JPEG]

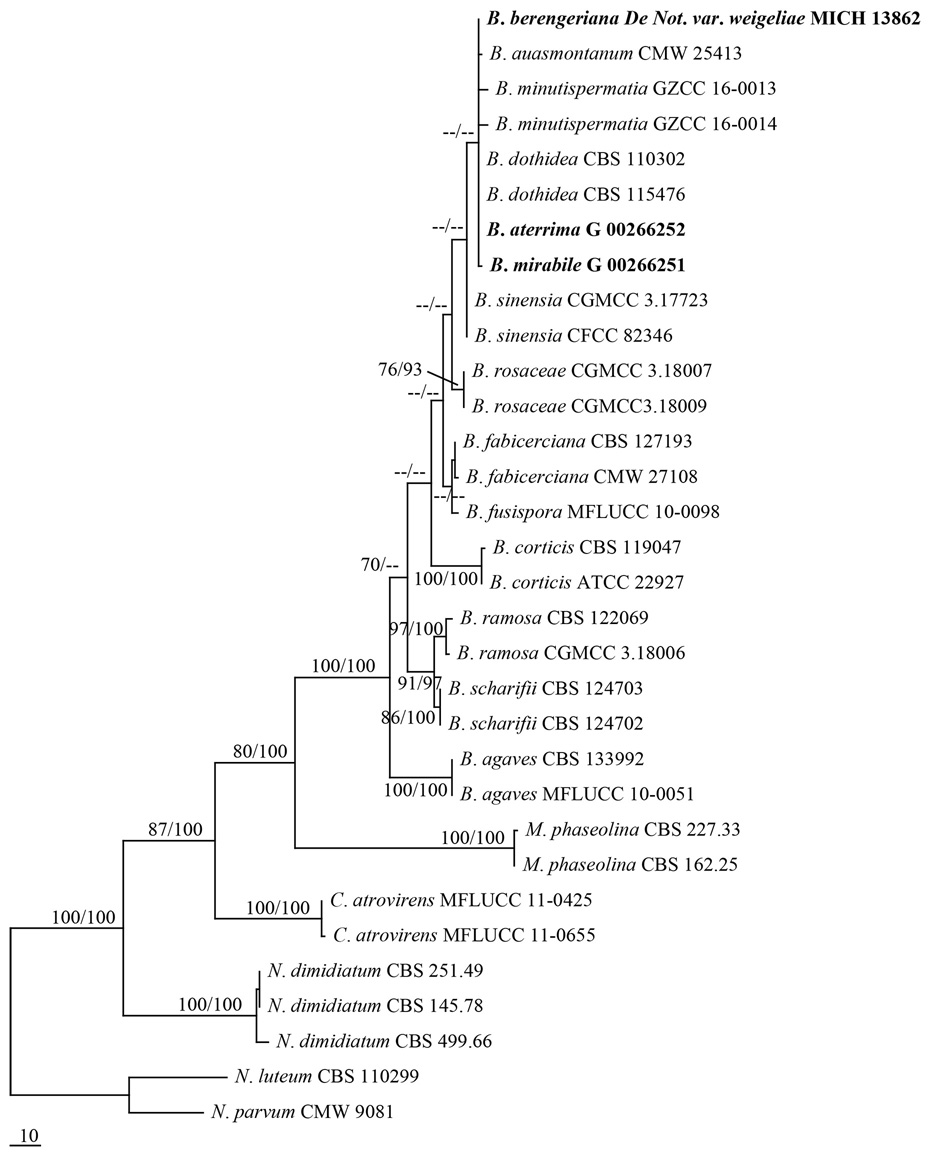

Supplement: Supplementary Figure 4 — One of the most parsimonious trees obtained from combined ITS, LSU, tub2, and tef1-a sequence data of Botryosphaeria spp. Outgroup taxa are Neofusicoccum luteum and Neofusicoccum parvum. Maximum parsimony (MP) support values above 70% and Bayesian posterior probabilities (PP) support above 80% are shown with MP bootstrap followed by Bayesian PP (MP/PP) values at the nodes. The species characterized in this study are in boldface. [file Image_4.JPEG]

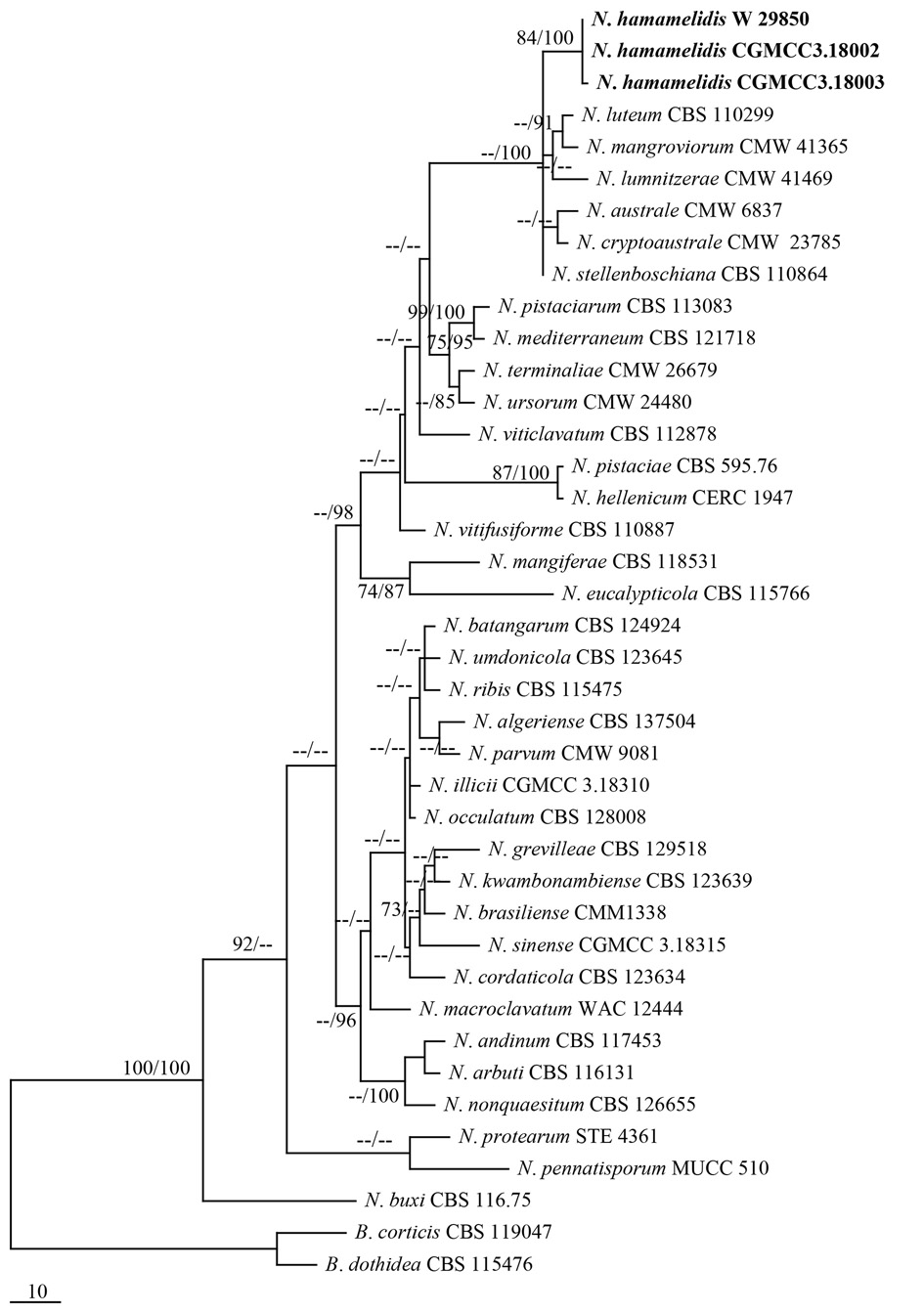

Supplement: Supplementary Figure 5 — One of the most parsimonious trees obtained based on combined ITS, tef1-α, and tub2 sequence data of Neofusicoccum spp. Outgroup taxon are Botryosphaeria dothidea and B. corticis. Maximum parsimony (MP) support values above 60% and Bayesian posterior probabilities (PP) support above 80% are shown with MP/PP, values at the nodes. The species characterized in this study are in boldface. [file Image_5.JPEG]

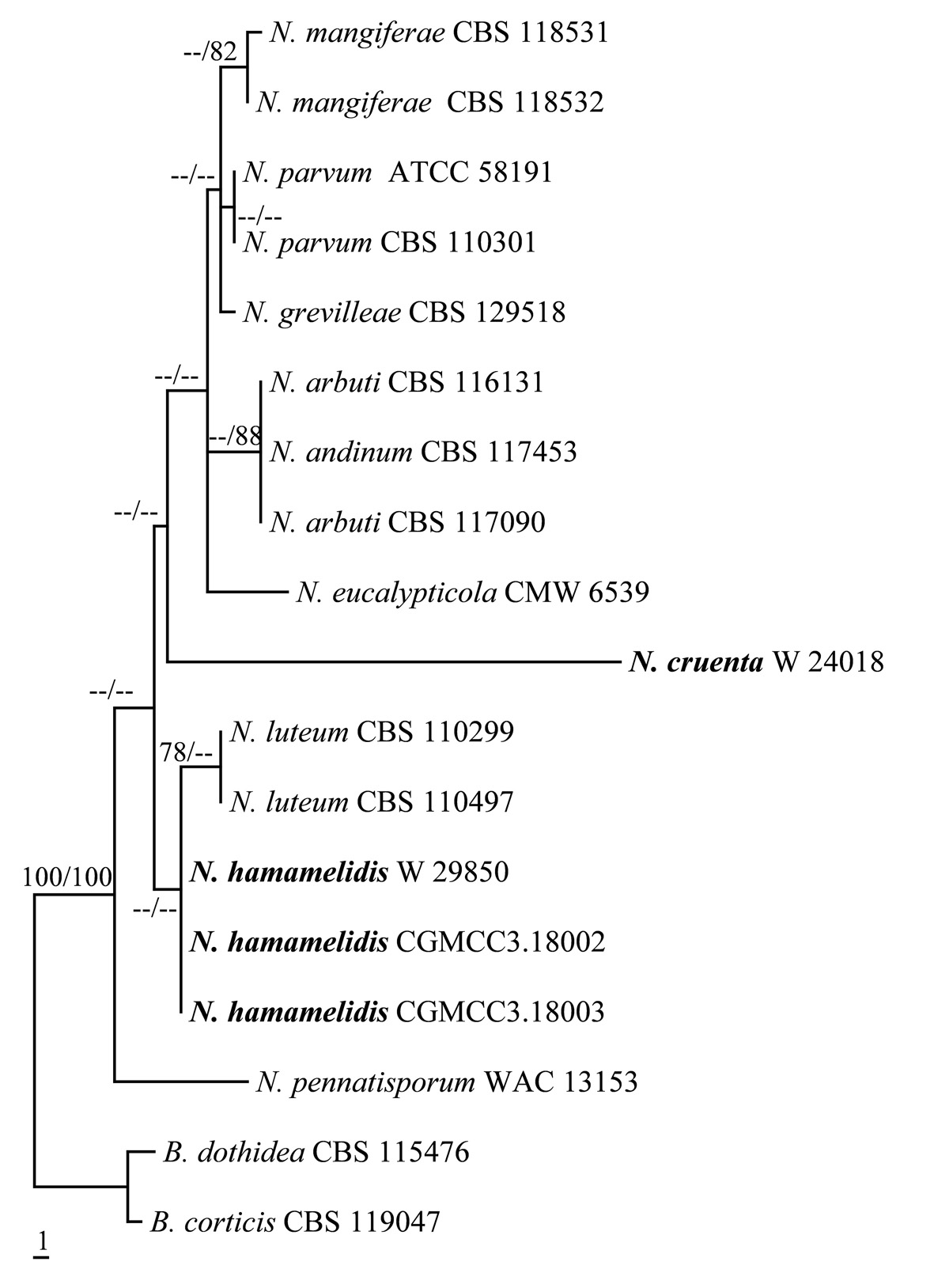

Supplement: Supplementary Figure 6 — One of the most parsimonious trees obtained from LSU sequence dataset of Neofusicoccum spp. Outgroup taxa are Botryosphaeria corticis and B. dothidea. Maximum parsimony (MP) support values above 70% and Bayesian posterior probabilities (PP) support above 80% are shown with MP bootstrap followed by Bayesian PP (MP/PP) values at the nodes. The species characterized in this study are in boldface. [file Image_6.JPEG]

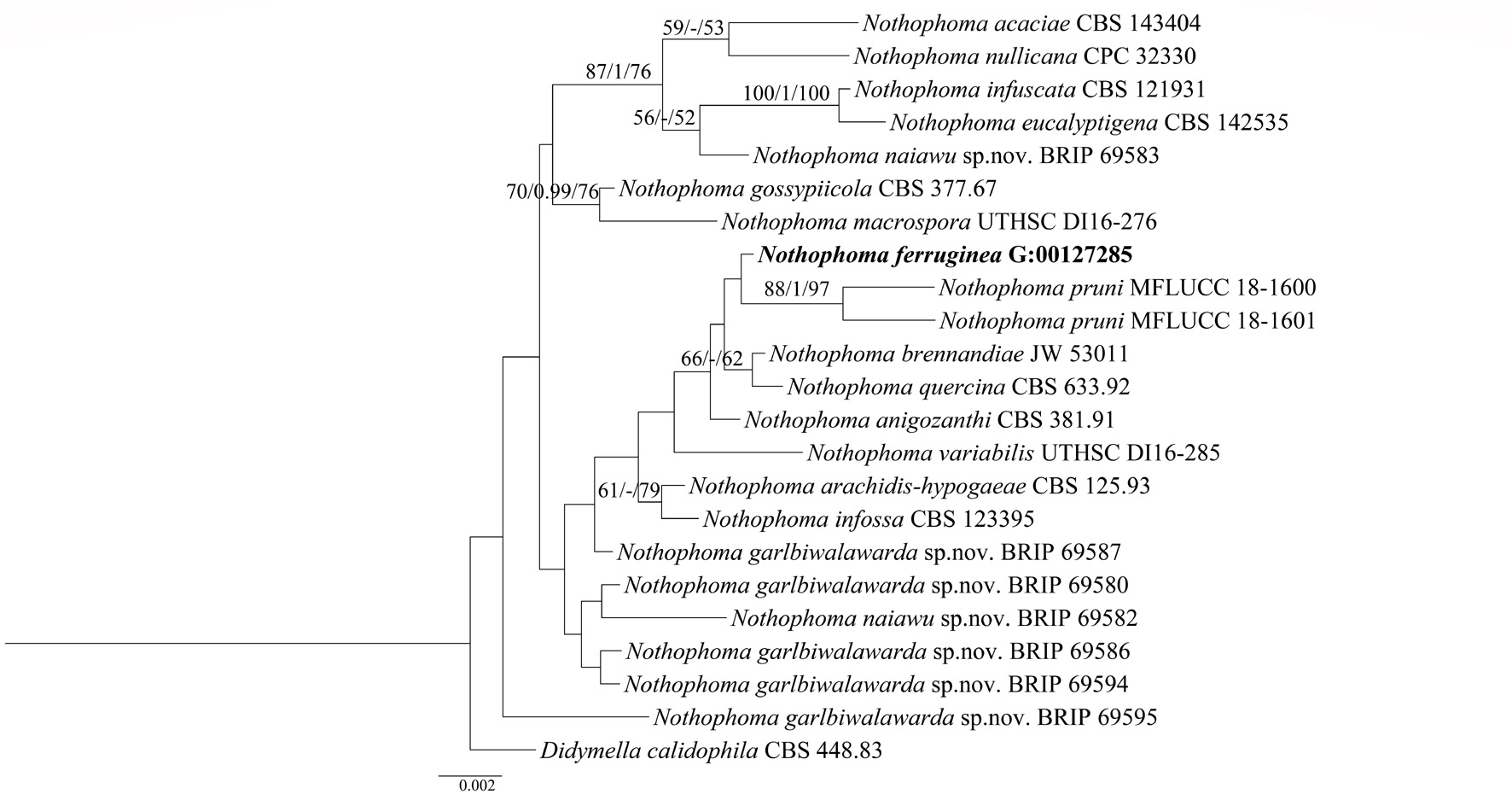

Supplement: Supplementary Figure 7 — One of the most parsimonious trees obtained from ITS and LSU sequence dataset of Nothophoma spp. Outgroup taxa is Didymella calidophila. Maximum likelihood (ML) support values above 50%, Maximum parsimony (MP) support values above 50%, and Bayesian posterior probabilities (PP) support above 95% are shown with ML and MP bootstrap followed by Bayesian PP (MP/PP/ML) values at the nodes. The species characterized in this study are in boldface. [file Image_7.JPEG]
