# Supplementary material for: Toward a Natural Classification of Botryosphaeriaceae: A Study of the Type Specimens of Botryosphaeria sensu lato
Source: Front Microbiol. 2021 Nov 3;12:737541. doi: 10.3389/fmicb.2021.737541 (PMC8595605; doi:10.3389/fmicb.2021.737541)
Supplement: Supplementary Table 1 — Species, specimens and GenBank accession numbers of sequences used in this study (newly generated sequences are indicated in bold). [file Table_1.DOCX]

Supplementary Table 1 Species, specimens and GenBank accession numbers of sequences used in this study (newly generated sequences are indicated in bold).

| Species | Culture/Specimen No. | | Host | Location | | | | GenBank accession numbers | | | | | | | | | | | | | | | |  |  |
| --- | --- | --- | --- | --- | --- | --- | --- | --- | --- | --- | --- | --- | --- | --- | --- | --- | --- | --- | --- | --- | --- | --- | --- | --- | --- |
|  |  |  |  |  |  |  |  | ITS | | LSU | | | | | *tef1-a* | | | | | | *tub2* | | |  |  |
| *Botryosphaeria agaves* | CBS 133992 ^T^ | | *Agave* sp. | Thailand | | | | JX646791 | | JX646808 | | | | | JX646856 | | | | | | JX646841 | | |  |  |
| *B*. *agaves* | MFLUCC 10-0051 | | *Agave* sp. | Thailand | | | | JX646790 | | JX646807 | | | | | JX646855 | | | | | | JX646840 | | |  |  |
| *B. auasmontanum* | CMW 25413^T^ | | *Pinus* sp. | Namibia | | | | KF766167 | | KF766332 | | | | | N/A | | | | | | N/A | | |  |  |
| *B. aterrima* | G 00266252^T^ | | *Ulmus* sp. | Germany | | | | **KU359182** | | **KU359187** | | | | | **KU359191** | | | | | | N/A | | |  | |
| *B*. *corticis* | CBS 119047 | | *Vaccinium corymbosum* | New Jersey, USA | | | | DQ299245 | | EU673244 | | | | | EU017539 | | | | | | EU673107 | | |  |  |
| *B*. *corticis* | ATCC 22927 | | *Vaccinium* sp. | North Carolina, USA | | | | DQ299247 | | EU673245 | | | | | EU673291 | | | | | | EU673108 | | |  |  |
| *B*. *dothidea* | CBS 115476 ^T^ | | *Prunus* sp. | Crocifisso, Switzerland | | | | AY236949 | | AY928047 | | | | | AY236898 | | | | | | AY236927 | | |  |  |
|  | CBS 110302 | | *Vitis vinifera* | [Montemor-o-Novo](http://zh.mindat.org/loc-132109.html), [Portugal](http://zh.mindat.org/loc-14425.html) | | | | AY259092 | | EU673243 | | | | | AY573218 | | | | | | EU673106 | | |  |  |
|  | MICH 13862 ^T^ | | *Wiegela sp.* | Russia | | | | **KY964329** | | N/A | | | | | N/A | | | | | | **KY964332** | | |  |  |
| *B*. *fabicerciana* | CBS 127193 ^T^ | | *Eucalyptus* sp. | Fujian, China | | | | HQ332197 | | N/A | | | | | HQ332213 | | | | | | N/A | | |  |  |
| *B*. *fabicerciana* | CMW 27108 | | *Eucalyptus* sp. | Fujian, China | | | | HQ332200 | | N/A | | | | | HQ332216 | | | | | | N/A | | |  |  |
| *B*. *fusispora* | MFLUCC 10-0098 ^T^ | | *Entada* sp. | Thailand | | | | JX646789 | | JX646806 | | | | | JX646854 | | | | | | JX646839 | | |  |  |
| *B*. *minutispermatia* | GZCC 16-0013 ^T^ | | dead wood | China | | | | KX447675 | | N/A | | | | KX447678 | | | | | | N/A | | | |  |  |
| *B*. *minutispermatia* | GZCC 16-0014 | | dead wood | China | | | | KX447676 | | N/A | | | | KX447679 | | | | | | N/A | | | |  |  |
| *B*. *mirabile* | G 0066251^T^ | | *Quercus* sp. | Switzerland, Genève | | | | **KU359181** | | **KU359186** | | | | N/A | | | | | | N/A | | | |  |  |
| *B*. *ramosa* | CBS 122069 ^T^ | | *Eucalyptus camaldulensis* | Bell Gorge, Australia | | | | EU144055 | | N/A | | | | EU144070 | | | | | | N/A | | | |  |  |
| *B*. *ramosa* | CGMCC3.18006 | | *Myrtaceae* | Hainan, China | | | | KX197072 | | KX197081 | | | | KX197092 | | | | | | KX197099 | | | |  |  |
| *B*. *rosaceae* | CGMCC3.18007 | | *Malus* sp. | Shandong, China | | | | KX197074 | | KX197083 | | | | KX197094 | | | | | | KX197101 | | | |  |  |
| *B*. *rosaceae* | CGMCC3.18009 | | *Malus* sp. | Henan, China | | | | KX197076 | | KX197085 | | | | KX197096 | | | | | | KX197103 | | | |  |  |
| *B*. *scharifii* | CBS 124703 ^T^ | | *Mangifera indica* | | Iran, Tehran | | | JQ772020 | | N/A | | | | JQ772057 | | | | | | N/A | | | |  |  |
| *B*. *scharifii* | CBS 124702 | | *Mangifera indica* | | Iran, Hormozgan | | | JQ772019 | | N/A | | | | JQ772056 | | | | | | N/A | | | |  |  |
| *B*. *sinensia* | CGMCC3.17723 | | *Morus* sp. | | Henan, China | | | KT343254 | | KX197090 | | | | KU221233 | | | | | | KX197107 | | | |  |  |
| *B*. *sinensia* | CFCC 82346 | | *Juglans regia* | | Beijing, China | | | KT343257 | | KX197091 | | | | KU221235 | | | | | | KX197109 | | | |  |  |
| *Cophinforma atrovirens* | MFLUCC  11-0425 ^T^ | | *Eucalyptus* sp. | | Thailand | | | JX646800 | | JX646817 | | | | JX646865 | | | | | | JX646848 | | | |  |  |
| *C*. *atrovirens* | MFLUCC 11-0655 | | *Eucalyptus* sp. | | Thailand | | | JX646801 | | JX646818 | | | | JX646866 | | | | | | JX646849 | | | |  |  |
| *Didymella calidophila* | CBS 448.83^T^ | | Desert soil | | Egypt | | | FJ427059 | | GU238052 | | | | – | | | | | | – | | | |  |  |
| *D*. *mutila* | CBS 112553 | | *Vitis vinifera* | | Portugal | | | AY259093 | | – | | | | – | | | | | | – | | | |  |  |
| *D*. *seriata* | CBS 112555 | | *Vitis vinifera* | | Portugal | | | AY259094 | | – | | | | – | | | | | | – | | | |  |  |
| *Macrophomina phaseolina* | CBS 227.33 | | *Zea mays* | | Unknown | | | KF531825 | | DQ377906 | | | | | | KF531804 | | | | | KF531806 | | | |  |
| *M*. *phaseolina* | CBS 162.25 | | *Eucalyptus* sp. | | Uganda | | | KF531826 | | DQ377905 | | | | | | KF531803 | | | | | KF531805 | | | |  |
| *Neofusicoccum* *algeriense* | CBS 137504 | | *Vitis vinifera* | | Algeria | | | KJ657702 | | – | | | | | | KJ657715 | | | | | KX505915 | | | |  |
| *N*. *andinum* | CBS 117453 ^T^ | | *Eucalyptus* sp. | | Venezuela | | | AY693976 | DQ377914 | | | | | | | AY693977 | | | | | N/A | | | |  |
| *N*. *arbuti* | CBS 116131 ^T^ | | *Arbutus menziesii* | | USA | | | AY819720 | DQ377915 | | | | | | | KF531792 | | | | | KF531793 | | | |  |
| *N*. *arbuti* | CBS 117090 | | *Arbutus menziesii* | | USA | | | – | DQ377919 | | | | | | | – | | | | | – | | | |  |
| *N*. *australe* | CMW 6837 ^T^ | | *Acacia* sp. | | Australia | | | AY339262 | | N/A | | | | | AY339270 | | | | | | AY339254 | | | |  |
| *N. batangarum* | CBS 124924 | | *Terminalia catappa* | | Africa | | | FJ900607 | | – | | | | | FJ900653 | | | | | | FJ900634 | | | |  |
| *N. brasiliense* | CMM 1338 | | *Mangifera indica* | | Brazil | | | JX513630 | | – | | | | | JX513610 | | | | | | KC794031 | | | |  |
| *N*. *buxi* | CBS 116.75 | | *Buxus sempervirens* | | France | | | KX464165 | | – | | | | | KX464678 | | | | | | N/A | | | |  |
| *N. cordaticola* | CBS 123634 | | *Syzygium* *cordatum* | | South Africa | | | EU821898 | | – | | | | | EU821868 | | | | | | N/A | | | |  |
| *N*. *cruenta* | W 1978-0010992 ^T^ | | *Polygonatum officinale* | | Czech Republic | | | N/A | | **KX197089** | | | | | N/A | | | | | | N/A | | | |  |
| *N. cryptoaustrale* | CMW 23785 | | *Eucalyptus* sp*.* | | South Africa | | | FJ752742 | | N/A | | | | | FJ752713 | | | | | | N/A | | | |  |
| *N. eucalypticola* | CBS 115766 | | *Eucalyptus* *rossii* | | Australia | | | AY615143 | | KF766368 | | | | | AY615135 | | | | | | AY615127 | | | |  |
| *N. eucalyptorum* | CMW 10125 | | *Eucalyptus grandis* | | South Africa | | | AF283686 | | – | | | | | AY236891 | | | | | | AY236920 | | | |  |
| *N. grevilleae* | CBS 129518 | | *Grevillea aurea* | | Australia | | | JF951137 | | JF951157 | | | | | N/A | | | | | | N/A | | | |  |
| *N. hellenicum* | | CERC 1947 | *Pistacia vera* | Greece | | | | KP217053 | | – | | | | | – | | | | | | – | | |  |  |
| *N*. *hamamelidis*^T^ | | W 29850 | *Hamamelidis virginiana* | Canada | | | | **KU359183** | | **KU359188** | | | | | **KU359192** | | | | | | **KX197112** | | |  |  |
| *N*. *hamamelidis* | | CGMCC3.18002 | *Larix gmelinii* | China | | | | **KU359184** | | **KU359189** | | | | | N/A | | | | | | **KX197110** | | |  |  |
| *N*. *hamamelidis* | | CGMCC3.18003 | *Larix gmelinii* | China | | | | **KU359185** | | **KU359190** | | | | | **KU359193** | | | | | | **KX197111** | | |  |  |
| *N. illicii* | | CGMCC 3.18310 | *Illicium verum* | Guangxi, China | | | | KY350149 | | – | | | | | – | | | | | | KY350155 | | |  |  |
| *N. kwambonambiense* | | CBS 123639 | *Syzygium* *cordatum* | South Africa | | | | EU821900 | | – | | | | | EU821870 | | | | | | EU821840 | | |  |  |
| *N. lumnitzerae* | | CMW 41469 | *Lumnitzera racemosa* | South Africa | | | | KP860881 | | – | | | | | KP860724 | | | | | | – | | |  |  |
| *N*. *luteum* | | CBS 110299 ^T^ | *Vitis vinifera* | Portugal | | | | AY259091 | | AY928043 | | | | | AY573217 | | | | | | DQ458848 | | |  |  |
| *N*. *luteum* | | CBS 110497 | Unknown | Portugal | | | | – | | EU673229 | | | | | – | | | | | | – | | |  |  |
| *N. macroclavatum* | | WAC 12444 | *Eucalyptus* *globulus* | Australia | | | | DQ093196 | | – | | | | | DQ093217 | | | | | | DQ093206 | | |  |  |
| *N. mangiferae* | | CBS 118531 | *Mangifera indica* | Australia | | | | AY615185 | | DQ377920 | | | | | DQ093221 | | | | | | AY615172 | | |  |  |
| *N*. *mangiferae* | | CBS 118532 | *Mangifera indica* | Australia | | | | – | | DQ377921 | | | | | – | | | | | | – | | |  |  |
| *N. mangroviorum* | CMW 41365 | | *Avicennia marina* | South Africa | | | | KP860859 | | | – | | | | | KP860702 | | | | | | – | |  |  |
| *N. mediterraneum* | CBS 121718 | | *Eucalyptus* sp. | Greece | | GU251176 | | | – | | | GU251308 | | | | | | GU251836 | | | | | |  |  |
| *N. nonquaesitum* | CBS 126655 | | *Umbellularia* | USA | | GU251163 | | | – | | | GU251295 | | | | | | GU251823 | | | | | |  |  |
| *N. occulatum* | CBS 128008 | | *Eucalyptus* *grandis hybrid* | Australia | | EU301030 | | | – | | | EU339509 | | | | | EU339472 | | | | | | |  |  |
| *N*. *parvum* | ATCC 58191 ^T^ | | *Populus nigra* | New Zealand | | AY236943 | | | AY928045 | | | AY236888 | | | | | AY236917 | | | | | | |  |  |
| *N*. *parvum* | CBS 110301 | | Unknown | Unknown | | AY259098 | | | AY928046 | | | AY573221 | | | | | EU673095 | | | | | | |  |  |
| *N*. *pennatisporum* | WAC 13153 ^T^ | | *Allocasuarina fraseriana* | Australia | | – | | | EF591942 | | | – | | | | | – | | | | | | |  |  |
| *N. pennatisporum* | MUCC 510 | | *Allocasuarina fraseriana* | Australia | | EF591925 | | | – | | | EF591976 | | | | | EF591959 | | | | | | |  |  |
| *N*. *pistaciae* | CBS 595.76 | | *Pistacia vera* | Greece | | KX464163 | | | – | | | KX464676 | | | | | | KX464953 | | | | | |  |  |
| *N*. *pistaciarum* | CBS 113083 | | *Pistacia vera* | USA | | KX464186 | | | – | | | KX464712 | | | | | | KX464998 | | | | | |  |  |
| *N. protearum* | STE-U 4361 | | *Protea magnifica* | South Africa | | | AF196295 | | – | | | | – | | | | | – | | | | | |  |  |
| *N. ribis* | CBS 115475 | | *Ribes* sp. | USA | | | AY236935 | | – | | | | AY236877 | | | | | AY236906 | | | | | |  |  |
| *N. sinense* | CGMCC 3.18315 | | Unknown | Guizhou,China | | | KY350148 | |  | | | | KY817755 | | | | | | KY350154 | | | | |  |  |
| *N*. *stellenboschiana* | CBS 110864 | | *Vitis vinifera* | South Africa | | | – | | – | | | | – | | | | | KX465047 | | | | | |  |  |
| *N*. *terminaliae* | CMW 26679 | | *Terminalia sericea* | South Africa | | | GQ471802 | | – | | | | GQ471780 | | | | | | KX465052 | | | | |  |  |
| *N. umdonicola* | CBS 123645 | | *Syzygium cordatum* | South Africa | | | EU821904 | | – | | | | EU821874 | | | | | EU821844 | | | | | |  |  |
| *N. ursorum* | CMW 24480 | | *Eucalyptus* *arboretum* | South Africa | | | FJ752746 | | – | | | | FJ752709 | | | | | – | | | | | |  |  |
| *N. viticlavatum* | CBS 112878 | | *Vitis vinifera* | South Africa | | | AY343381 | | – | | | | AY343342 | | | | | – | | | | | |  |  |
| *N. vitifusiforme* | CBS 110887 | | *Vitis vinifera* | South Africa | | | AY343383 | | – | | | | AY343343 | | | | | – | | | | | |  |  |
| *Neoscytalidium hyalinum* | CBS 499.66 | | *Mangifera indica* | Unknown | | | KF531820 | | DQ377925 | | | | KF531798 | | | | | KF531800 | | | | | |  |  |
| *Neos*. *hyalinum* | CBS 251.49 | | *Juglans regia* | USA | | | | KF531819 | DQ377923 | | | | KF531797 | | | | | KF531799 | | | | | |  |  |
| *Neos*. *hyalinum* | CBS 145.78 ^T^ | | *Homo sapiens* | UK | | | | KF531816 | DQ377922 | | | | KF531795 | | | | | KF531796 | | | | | |  |  |
| *Nothophoma acaciae* | CBS 143404 ^T^ | | *Acacia melanoxylon* | Australia | | | | MG386056 | MG386109 | | | | – | | | | | – | | | | | |  |  |
| *No. anigozanthi* | CBS 381.91; PD 79/1110 | | *Anigozanthus maugleisii* | The Netherlands | | | | GU237852 | GU238039 | | | | – | | | | | – | | | | | |  |  |
| *No. arachidis-hypogaeae* | CBS 125.93; PD 77/1029 | | *Arachis hypogaea* | India | | | | GU237771 | GU238043 | | | | – | | | | | – | | | | | |  |  |
| *No. brennandiae* | CBS 145912 ^T^ | | *Soil* | The Netherlands | | | | MN823579 | MN823430 | | | | – | | | | | – | | | | | |  |  |
| *No. eucalyptigena* | CBS 142535 ^T^ | | *Eucalyptus* sp. | | Australia | | | KY979771 | | KY979826 | | | | | | – | | | | | – | |  |  |  |
| *No. ferruginea* | G 00127285 ^T^ | | *Alnus glutinosa* | | Switzerland | | | **KY964330** | | **KY964331** | | | | | | N/A | | | | | N/A | |  |  |  |
| *No. garlbiwalawarda* | BRIP 69580 | | *Senna artemisioides* | | Australia, SA, Adelaide | | | MN5676782 | | – | | | | | | – | | | | | – | |  |  |  |
|  | BRIP 69586 | | *Senna artemisioides* | | Australia, SA, Berri | | | MN5676783 | | – | | | | | | – | | | | | – | |  |  |  |
|  | BRIP 69587 | | *Senna artemisioides* | | Australia, SA, Berri | | | MN5676784 | | – | | | | | | – | | | | | – | |  |  |  |
|  | BRIP 69584 | | *Senna artemisioides* | | Australia, SA, Kimba | | | MN5676785 | | – | | | | | | – | | | | | – | |  |  |  |
|  | BRIP 69585 ^T^ | | *Senna artemisioides* | | Australia, SA, Wudinna | | | MN5676786 | | – | | | | | | – | | | | | – | |  |  |  |
| *No. gossypiicola* | CBS 377.67 | | *Gossypium* sp. | | USA, Texas | | | GU237845 | | GU238079 | | | | | | – | | | | | – | |  |  |  |
| *No. infossa* | CBS 123395^T^ | | *Fraxinus pennsylvanica* | | Argentina | | | FJ427025 | | GU238089 | | | | | | – | | | | | – | |  |  |  |
| *No. infossa* | CBS 123394 | | *Fraxinus pennsylvanica* | | Argentina | | | FJ427024 | | GU238088 | | | | | | – | | | | | – | |  |  |  |
| *No. infuscata* | CBS 121931 ^T^ | | *Acacia longifolia* | | New Zealand | | | MN973559 | | MN943766 | | | | | | – | | | | | – | |  |  |  |
| *No. macrospora* | UTHSC DI16-199 ^T^ | | *Homo sapiens* | | USA, Arizona | | | LN880536 | | NG_069482 | | | | | | – | | | | | – | |  |  |  |
| *No. naiawu* | BRIP 69583 ^T^ | | *Senna artemisioides* | | Australia, SA, Blanchetown | | | MN5676787 | | – | | | | | | – | | | | | – | |  |  |  |
|  | BRIP 69582 ^T^ | | *Senna artemisioides* | | Australia, SA, Blanchetown | | | MN5676788 | | – | | | | | | – | | | | | – | |  |  |  |
| *No. nullicana* | CPC 32330 ^T^ | | *Acacia falciformis* | | Australia | | | NR_156665 | | – | | | | | | – | | | | | – | |  |  |  |
| *No. pruni* | MFLUCC 18-1600 | | *Prunus avium* | | China | | | MH827007 | | MH827028 | | | | | | – | | | | | – | |  |  |  |
|  | MFLUCC 18-1601 | | *Prunus avium* | | China | | | MH827005 | | MH827026 | | | | | | – | | | | | – | |  |  |  |
| *No. quercina* | CBS 633.92; ATCC 36786; VKM MF-325 | | *Microsphaera alphitoides from Quercus* sp. | Ukraine | | | | GU237900 | EU754127 | | | | – | | | | | – | | | | | |  |  |
| *No. variabilis* | UTHSC DI16-285 ^T^ | | *Homo sapiens* | USA | | | | LT592939 | – | | | | – | | | | | – | | | | | |  |  |

* N/A: no sequence available in GenBank.

–: not used in this study.

^T^: Type specimens and ex-type cultures.
